# Natural Background and the Anthropogenic Enrichment of Mercury in the Southern Florida Environment: A Review with a Discussion on Public Health

**DOI:** 10.3390/ijerph21010118

**Published:** 2024-01-22

**Authors:** Thomas M. Missimer, James H. MacDonald, Seneshaw Tsegaye, Serge Thomas, Christopher M. Teaf, Douglas Covert, Zoie R. Kassis

**Affiliations:** 1 U. A. Whitaker College of Engineering, Florida Gulf Coast University, 10501 FGCU Boulevard South, Fort Myers, FL 33965-6565, USA; zkassis@fgcu.edu; 2 Environmental Geology Program & Honors College, Florida Gulf Coast University, 10501 FGCU Boulevard South, Fort Myers, FL 33965-6565, USA; jmacdona@fgcu.edu; 3 Department of Bioengineering, Civil and Environmental Engineering, Florida Gulf Coast University, 10501 FGCU Boulevard South, Fort Myers, FL 33965-6565, USA; stsegaye@fgcu.edu; 4 Department of Ecology and Environmental Studies, Florida Gulf Coast University, 10501 FGCU Boulevard South, Fort Myers, FL 33965-6565, USA; sthomas@fgcu.edu; 5 Institute for Science & Public Affairs, Florida State University, Tallahassee, FL 32310, USA; cteaf@hswmr.com; 6 Hazardous Substance & Waste Management Research, 2976 Wellington Circle West, Tallahassee, FL 32309, USA; dcovert@hswmr.com

**Keywords:** Hg, southern Florida, soils, geologic units, Everglades, groundwater, atmospheric deposition, exposure, public health risk

## Abstract

Mercury (Hg) is a toxic metal that is easily released into the atmosphere as a gas or a particulate. Since Hg has serious health impacts based on human exposure, it is a major concern where it accumulates. Southern Florida is a region of high Hg deposition in the United States. It has entered the southern Florida environment for over 56 MY. For the past 3000 to 8000 years, Hg has accumulated in the Everglades peatlands, where approximately 42.3 metric tons of Hg was deposited. The pre-industrial source of mercury that was deposited into the Everglades was from the atmosphere, consisting of combined Saharan dust and marine evasion. Drainage and the development of the Everglades for agriculture, and other mixed land uses have caused a 65.7% reduction in the quantity of peat, therefore releasing approximately 28 metric tons of Hg into the southern Florida environment over a period of approximately 133 years. Both natural and man-made fires have facilitated the Hg release. The current range in mercury release into the southern Florida environment lies between 994.9 and 1249 kg/yr. The largest source of Hg currently entering the Florida environment is from combined atmospheric sources, including Saharan dust, aerosols, sea spray, and ocean flux/evasion at 257.1–514.2 kg/yr. The remobilization of Hg from the Everglades peatlands and fires is approximately 215 kg/yr. Other large contributors include waste to energy incinerators (204.1 kg/yr), medical waste and crematory incinerators (159.7+ kg/yr), and cement plant stack discharge (150.6 kg/yr). Minor emissions include fuel emissions from motorized vehicles, gas emissions from landfills, asphalt plants, and possible others. No data are available on controlled fires in the Everglades in sugar farming, which is lumped with the overall peatland loss of Hg to the environment. Hg has impacted wildlife in southern Florida with recorded excess concentrations in fish, birds, and apex predators. This bioaccumulation of Hg in animals led to the adoption of regulations (total maximum loads) to reduce the impacts on wildlife and warnings were given to consumers to avoid the consumption of fish that are considered to be contaminated. The deposition of atmospheric Hg in southern Florida has not been studied sufficiently to ascertain where it has had the greatest impacts. Hg has been found to accumulate on willow tree leaves in a natural environment in one recent study. No significant studies of the potential impacts on human health have been conducted in southern Florida, which should be started based on the high rates of Hg fallout in rainfall and known recycling for organic sediments containing high concentrations of Hg.

## 1. Introduction

Mercury (Hg) is a naturally occurring element that can have major environmental and human health impacts [1,2,3,4,5,6,7,8,9,10]. It is ubiquitous in gaseous and particulate form in the atmosphere and has concentrations at similar ranges over continental-scale areas of the Earth’s near-surface [11]. Complex chemical and biochemical interactions between the atmosphere and the land masses tend to control localized Hg concentrations in the surface biosphere and oceans [12,13,14]. Due to its chemical properties, Hg is readily transported for great distances in the atmosphere, which makes it both a local and global problem. Three forms of Hg are emitted into the atmosphere, and include elemental Hg (Hg^o^), reactive gaseous Hg (RGM), and particulate Hg (PHg). Of these three, Hg^o^ constitutes >60% of atmospheric Hg and has a residence time of 1 to 2 years [15]. The other forms of Hg tend to be deposited closer to their sources.

Both natural and anthropogenic sources of Hg emission to the atmosphere have varied greatly over time. Natural contributions of Hg to the atmosphere come primarily from volcanos, geothermal vents, and point sources, some of which are Hg-bearing mineral deposits occurring at the land surface [16,17,18,19,20,21]. Additional natural Hg sources include geologically enriched soils [22,23], coal outcrops, biomass fires [7,11,24,25,26,27,28,29], and diffusion from seas and oceans [13,30,31,32,33,34,35]. Gustin [20] suggests that the contribution of Hg from natural materials and systems has been underestimated in many locations. Variation in Hg concentrations in the rock record before anthropogenic influence suggests that volcanism was the key factor controlling enriched strata in ancient sedimentary rocks [21,36,37,38,39].

The anthropogenic origins of Hg range from major sources, such as coal-fired and oil-fired power plants [40,41,42,43], iron-steel manufacturing [42], non-ferrous metal smelters [44,45], caustic soda production [11,42], cement production [11,42,46], coal-bed fires [47], industrial municipal waste to energy, and biomedical waste incinerators [48]. More localized sources include landfills containing municipal and industrial waste [11,49,50,51,52], wastewater treatment plant air outflow [11,53], wastewater sludge applied to fields [54], mine waste [55,56], man-created forest fires (see references in biomass fires section), and mercury and gold mining [45].

The atmospheric deposition of Hg occurs both in wet and dry forms and is a global phenomenon [14,57]. Three types of Hg are found in the atmosphere, including gaseous elemental Hg (GEM), gaseous oxidized Hg (GOM), and particle-bound Hg (PBM) [58]. GOM has an extensive atmospheric lifetime with long transport but is generally insoluble and inert [59]. GOM is more associated with local or regional Hg emissions and has a shorter atmospheric residence time [60]. The formation of GOM can be enhanced by higher temperatures and photochemical activity related to the ozone-induced oxidation of GEM [61]. Most of the total dry mercury deposition on water, soil, and plants is GOM and PBM, whereas GEM is a minor contributor [61,62,63].

**Figure 1:**
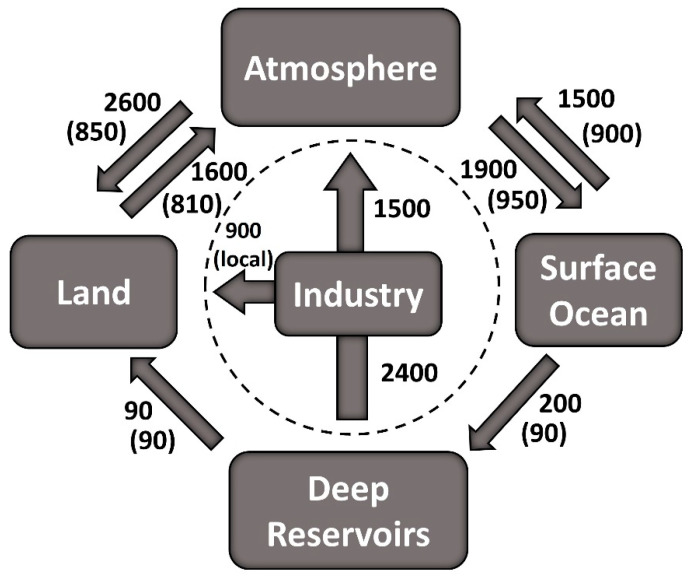
Global geochemical cycle of Hg showing approximated values for pre-industrial and modern times (modified from values compiled in Mason and Sheu [33] as adapted in Schwartzendruber and Jaffee [71]). The values shown in the figure are in tons per year. The pre-industrial values are contained within the parentheses.

The concentration of Hg in the atmosphere is controlled by a combination of complex natural biogeochemical processes in combination with variation in the inputs of both natural and anthropogenic sources of Hg [64,65,66,67,68,69]. Anthropogenic gaseous elemental Hg (GEM) emissions have significantly impacted the balance of inputs to and outputs from the atmosphere. Concentrations of GEM have been estimated to have increased by a factor of 3 ± 1 from pre-industrial to modern times based on several studies [14,70]. Mason and Sheu [33] developed a simplified global geochemical cycle for mercury with a comparison of pre-industrial versus modern times (Figure 1). It can be clearly observed that industrial activity impacts have greatly mobilized Hg transport into the environment.

A study of two Pyrenean peat bogs in southwestern Europe suggests a higher ratio from Holocene to modern times. Enrico et al. [72] studied two peat bogs, the first of which had a 10,000-year record of sphagnum moss deposition (Pinet peat) and a second bog was a high-altitude sphagnum moss deposit (Estibere peat). Both peat bogs are located in an area of minimal industrial activity and were not subject to intensive anthropogenic disturbance. The results of this study indicate that the mean GEM concentration during the Middle Ages (800–1550 CE) was 0.38 ± 0.08 ng m^−3^ compared to 1.0 ± 0.2 ng m^−3^ during pre-industrial times (1550–1780 CE) with a maximum concentration at 3.9 ± 0.5 ng m^−3^ occurring between 1946 and 1967, and a leveling off to 1.5 ± 0.6 ng m^−3^ from 1990–2011 [72]. They also found the background concentration during the Holocene to be 0.27 ± 0.11 ng m^−3^, which equates to a 14.4 ratio between the Holocene and the maximum concentration value. The deposition rates obtained from the age-dated cores are given in Table 1. It should be noted that the deposition is predominantly dry (approximately 80%).

Common human exposure to Hg is by directly breathing atmospheric Hg or secondarily from ingestion of food [73,74,75]. Perhaps the most important event that highlighted the need to control discharges of Hg was the sickness and death that resulted from eating contaminated fish from Minamata Bay, Japan. Between 1953 and 1970, 121 people were poisoned, resulting in 46 deaths. Another village in the area reported 47 cases and six deaths. Nelson et al. [76] reported that the sickness and death was caused primarily by intake of methylmercury. Lambert et al. [77] found that methylmercury can biomagnify up to 1 million times in the aquatic food web. In the United States, high concentrations of Hg were found in various species of freshwater fish, which resulted in restrictions on sport and commercial fishing in 18 states [78]. Because of the discovery of Hg in canned tuna and in swordfish, experts from the U.S. Food and Drug Administration suggested that certain types of fish should not be eaten until further research demonstrated what safety precautions should be taken. An active review of Hg and a series of other toxic compounds was initiated by the U.S. Environmental Protection Agency (USEPA) beginning in 1970 [79]. A series of recommendations on the use of Hg and a variety of pesticides was published in 1971 [80]. Actions that led to the denial of registration of various Hg-containing products started the control of Hg discharge into the environment.

In the FDA Modernization Act of 1972, an assessment of Hg in foods and drugs was mandated in the United States. The estimated global emissions of anthropogenic mercury in 1995 was approximately 1900 metric tons [43]. This quantity estimate helped guide concerted global regulatory efforts to reduce the quantity of mercury emissions to the atmosphere. In 2004, the FDA began to issue guidance on the maximum amount of fish containing Hg that should be consumed in the diet of adults and children. In 1997, the USEPA [81] issued a report to the Congress of the United States on atmospheric emissions of Hg. From 2004 to 2006, a series of reports were issued by the USEPA in support of the Clean Air Mercury Rule [82,83,84,85]. As efforts continued to limit Hg emissions to the environment, the USEPA released a health assessment for Hg exposure [86] and an updated inventory of Hg emissions [87]. Following a global assessment of atmospheric Mg conducted by the United Nations Environmental Global Mercury Partnership, 140 nations adopted the Minamata Convention on Mercury on 16 November 2013 [88,89]. The primary goal of the Minamata Convention was to reduce the atmospheric emissions of Hg for the purpose of reducing impacts to human health [90].

Thus, with the global and more specific United States perspective as the background, the purpose of this paper is to provide a review of Hg in the environment of southern Florida and its potential impacts on public health. Southern Florida is particularly relevant since it has only been recently industrialized, but mostly in the form of population migration and the associated infrastructure (e.g., electric power production, cement production, waste disposal). Historically, it has not been the location of a high density of heavy industrial facilities associated with high atmospheric emissions of Hg. However, southern Florida contains a very large natural reservoir of legacy Hg in the peatlands of the Everglades that has been impacted by atmospheric deposition of Hg for thousands of years [91,92]. While many peatlands around the world also contain high concentrations of Hg [72,93,94,95,96], perhaps the Everglades peatland is unique in that it is extensively farmed in some areas, contains an extensive drainage system, sugar cane crops are burned on a seasonal basis, and the natural areas are subject to dry season fires. These processes tend to recycle, recirculate, and discharge Hg to surrounding and downwind environments. Because of therelatively high concentrations of Hg found in the Florida environment, the Florida Atmospheric Mercury Study (FAMS) was conducted to measure Hg at seven stations across Florida at different times between 1992 and 1996 [97,98,99]. Based on numerous studies of Hg in the southern Florida environment, the addition of anthropogenic Hg and the recycling of natural Hg deposited in soils has impacted plants, animals (in particular fish), and public health to variable degrees. An inventory of sources of Hg in southern Florida has been compiled and discussed with the purpose of defining what is known, what is not known, and what research should be conducted to limit future impacts on public health in southern Florida.

## 2. Geochemistry of Mercury

### 2.1. Chemical Properties

Hg is the only metal that is a liquid at room temperature and has a set of rather unique chemical properties (Table 2). Although it has a density 13 times greater than water, it has a rather high vapor pressure, which helps make it ubiquitous in the atmosphere. Hg (Hg^o^) has rather low solubility in water at 50 μg/L at 25 °C, so it has a more robust preference for atmospheric transport [100]. The ionic states of +1 and +2 are more common in water compared to the atmosphere [60].

Hg occurs as a number of chemical species depending upon whether the occurrence is in the atmosphere or within water. Elemental Hg (Hg^o^) occurs both in the atmosphere and in water. However, the most common form is gaseous elemental Hg (GEM) in the atmosphere. Natural surface water is nearly always saturated or supersaturated with elemental Hg with respect to atmospheric Hg concentrations [101]. Divalent Hg (HgII) occurs in inorganic and organic forms under gaseous, dissolved, and solid states. The toxic methylated forms of Hg are of considerable interest because of biologic cycling and accumulation by living organisms [102]. Dimethylmercury (DMHg) is a form that is considered to be highly toxic based on its ability to absorb into the human gut tissue with a tendency to bioaccumulate [71]. Monomethylmercury is also much more toxic compared to H^o^ due to its rapid gut absorption and toxicological properties [103]. The total airborne Hg is the sum of Hg^o^, Hg(II), and particulate Hg (PHg). Waterborne Hg is rather complex with some portioning between DMHg + Hg^o^, Hg(II), and MMhg+ PHg [71].

### 2.2. Oxidation–Reduction Reactions in the Atmosphere

**Figure 2:**
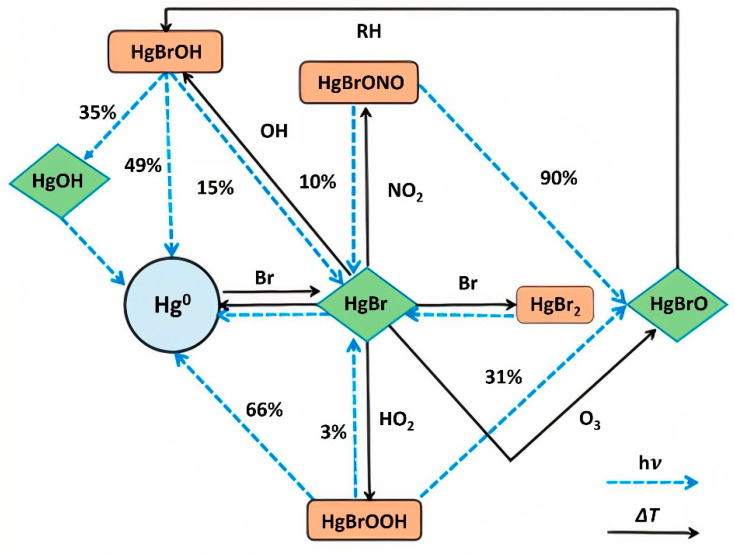
A description of the thermal and photochemical reactions of mercury in the atmosphere (modified from Saiz-Lopez et al. [38], republished with permission).

Within the atmosphere, a series of reactions impact the concentration of H^o^ and residence times of various forms of Hg [38,104]. While g enters the atmosphere primarily in the elemental form H^o^, it is oxidized to the more reactive forms Hg(I) and Hg(II). The photoreduction of Hg(I) and Hg(II) tends to offset the efficiency of bromine Hg^o^ oxidation, thereby matching the observed atmospheric variability of Hg^o^ and the observed residence time of 3 to 6 months [38]. The thermal oxidation of Hg also plays an important role in the atmospheric chemistry of Mg, which has a very complex cycle (Figure 2). This recent research suggests that even the best models of Hg chemical cycling in the atmosphere contain great uncertainty and will require more data to produce better estimates for residence time and predicted concentrations of the various mercury types in different regions [38,105,106].

### 2.3. Oxidation Reactions in Surface Ocean Water and Other Freshwater Bodies

Since approximately one third of the Hg flux to the atmosphere occurs from the oceans [33], it is important to understand the kinetics of dissolved gaseous Hg (DGM) and what factors influence the rates of release [32,107]. Field experiments and modeling were conducted by Qureshi et al. [108] to assess the pathways of oceanic mercury to atmospheric Hg. Photooxidation–photoreduction was found to not be a simple reversible reaction but involves an immediate reaction to a third form of Hg. The range in measured pseudo-first-order kinetic rate constants was in the range of 0.15–0.93 h^−1^. They concluded that microbes and colloids did not significantly impact the rate constants. This conclusion differs from some research performed in freshwater lakes wherein microbial reduction is considered to be an important process [109].

### 2.4. Oxidation–Reduction Reactions in Soils and Bioavailability of Mercury

Redox reactions in soils and freshwater systems, including lakes and wetland areas, play an important role in the toxicity of Hg and how it cycles in these environments. These processes are biochemical in nature and occur under a variety of natural and man-altered conditions. These processes play a major role in Hg transport and type in the southern Florida environment [110] (see Section 7.4).

Lakes and wetland areas commonly have three sources of methylmercury, including internal production, inputs from the watershed, and atmospheric wet and dry fallout [111]. One key process is the methylation of Hg, which commonly occurs in living organic material and soils and can be exacerbated by fertilization of the soil [112]. An investigation of Hg reduction and complexation by natural organic matter in anoxic environments showed that Hg(II) can be reduced to H^o^ via a thiolate ligand-induced oxidation complexation process [113]. Further, with a low concentration of humic acid of 0.2 mg/L, Hg(II) can be readily reduced to Hg^o^, but under higher concentrations the reduction process is inhibited. Gu et al. [113] also concluded that the complexation of Hg in anoxic sediment and water both influences the Hg speciation and biological uptake that leads to the formation of methylmercury. Natural organic matter (NOM) actually plays a dual role by simultaneously reducing and oxidizing Hg through two different mechanisms [114]. The reduction of Hg(II) is caused by reduced state quinones and oxidation is caused by low molecular weight thiol compounds [115]. Therefore, the rather rapid (two to six times faster than the photochemical reaction in surface water) production of reactive Hg enhances microbial uptake and methylation in anoxic environments [114].

In some wetland areas, other processes can affect the rate of Hg methylation. In an artificially maintained wetland area in southern Florida, Feng et al. [110] found that the wetting and drying cycles and the influx of sulfate in surface water enhanced the rate of Hg methylation. This is a two-step process in which the sulfate increased in concentration, which increased the occurrence of sulfate-reducing bacteria, ultimately increasing the rate of Hg methylation. Feng et al. [110] recommended the elimination of the drying process to reduce the methylation rate. Other research has suggested that upland soils can also contribute to Hg methylation during water saturation, which creates an anoxic condition conducive to the growth of typical Hg-methylating bacteria, including *Clostridium*, *Acetonema*, and *Geobacter* [116].

## 3. Overview of Natural Global Occurrence of Mercury in Rocks and Soils with Emphasis on Carbonate Rocks and Sediments

Inorganic Hg in the environment is described in detail in Fleischer et al. [117]. The Hg content of igneous rocks is generally <200 μg/kg with an average of approximately 100 μg/kg. Deep mantle rocks can have concentrations in the hundreds of μg/kg. Sedimentary rocks contain mercury concentrations of <200 μg/kg with the exception of shales, coals, organic-rich clays, and organic-rich soils. High concentrations have been found in some petroleum (California).

Hg is classified as a chalcophilic (low oxygen affinity, high sulfur affinity) element and tends to occur in sulfides [117]. The most common Hg-containing minerals are the sulfides cinnabar and metacinnabar (HgS), and native Hg. Hg formed from hydrothermal processes can commonly crystalize as corderoite (Hg_3_S_2_Cl_2_), livingstonite (HgSb_4_S_7_), and wartzite ([HgCuFe]_12_Sb_4_S_13_) [118]. The minor cationic substitution of Hg can also occur in other sulfides such as schwartzite, sphalerite, and wurtzite [118]. The U.S. Geological Survey reported the concentration of Hg in a variety of rock types from the literature [119]. While the various igneous and metamorphic rock types are not relevant to southern Florida, they do occur in source areas of Saharan dust that does impact southern Florida.

For comparative purposes to geologic data for Florida, a compilation of Hg concentration measurements in carbonate rocks has been made (Table 3). Although these data have been published within the context of peer-review, the analytical methods used to measure Hg concentrations in the past have been improved considerably since some of these measurements were made. Because of the difficulty in quantifying Hg in sediments in the past, it was commonly omitted from the list of heavy metals measured.

## 4. Naturally Occurring Hg Concentrations in Geological Units and Soils of Southern Florida

### 4.1. Hg Occurrence in Major Stratigraphic Units (Miocene to Early Eocene) in Southern Florida

Hg concentrations occurring in the Miocene to Lower Eocene sediments of southern Florida and some adjacent counties have been measured by the Florida Geological Survey (Table 4). With the exception of the Hawthorn Group (late Oligocene to Middle Miocene), these are primarily carbonate sediments with limestones being dominant in the Suwannee Limestone and Ocala Group (Late Eocene) and dolostones in the Avon Park Formation (Middle Eocene). The Hawthorn Group sediments are a mix of siliciclastics with clay and carbonates. The overall average Hg concentration for all 46 analyses is 15.3 μg/kg. The average concentrations of Hg in the four formations are as follows: Hawthorn Group at 23.6 μg/kg, the Suwannee Limestone at 17.3 μg/kg, the Ocala Group at 6.9 μg/kg, and the Avon Park Formation at 21.1 μg/kg. In comparison with the mercury concentrations measured in older carbonate rocks (Table 3), these values are generally lower.

**Figure 3:**
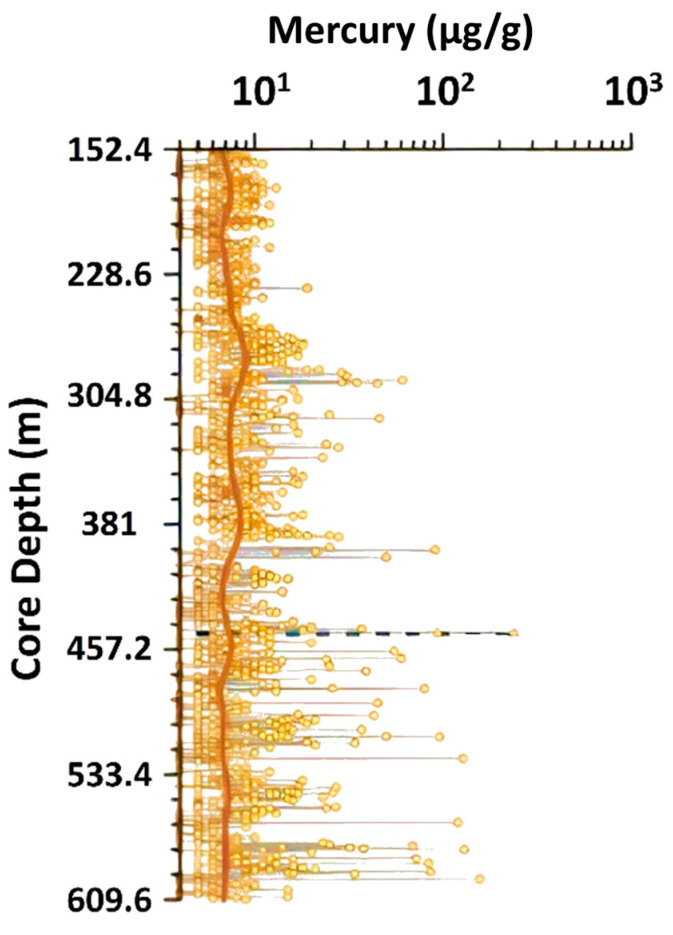
Hg measurements made in core L-63N north of Lake Okeechobee in μg/g (from MacDonald et al. [130,131]). Graph scale is semilogarithmic. The yellow points are individual measurements and the red line is the running average.

**Figure 4:**
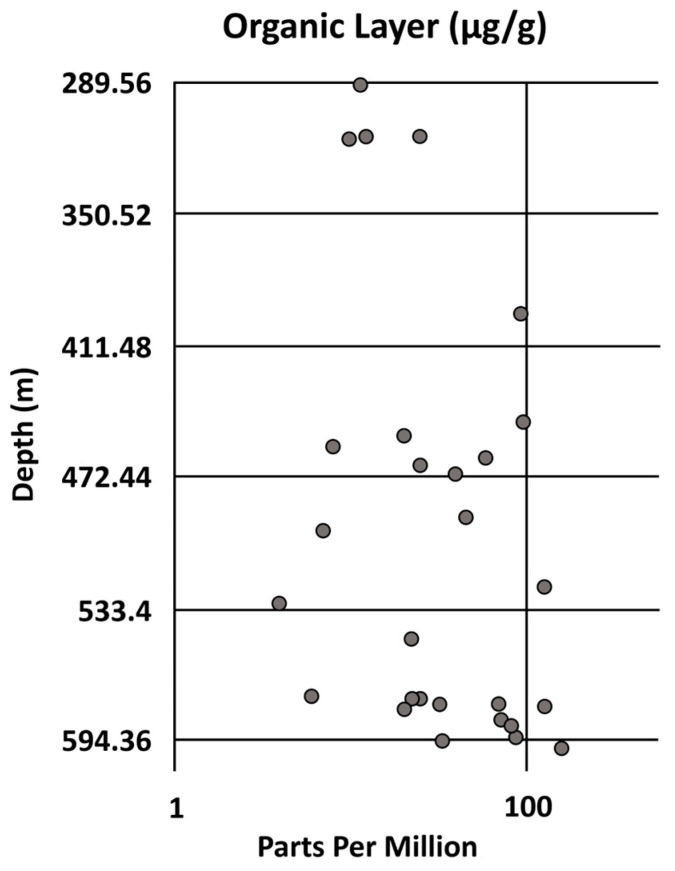
Occurrence of Hg in organic layers within core L-63N from MacDonald et al. [130,131]. Most organic layers occur in the Avon Park Graph and Oldsmar Formations. Scale is semilogarithmic.

A recent investigation was conducted by MacDonald et al. [130,131] on trace metals in a continuous core that penetrated approximately 457 m into the Miocene to Lower Eocene stratigraphic section of southern Florida. The site of the core was just north of Lake Okeechobee along Taylor Creek (Canal L63). Measurements were made approximately every 30 cm using a calibrated portable X-ray fluorescence (XRF) unit (Figure 3). The Hg concentrations had a large range in values and were found above detection limits in 1177 of the 1338 measurements made. The detection limit for Hg was 1 μg/g [130]. The range in concentration above the detection limit was 4 to 237 μg/g with an average of 9.91 μg/g. The highest values were located in the Avon Park Formation in the deeper part of the core. There was a strong correlation between high concentrations of Hg and the occurrence of organic layers ([130,131]; Figure 4). Four of these organic layers were located in the Ocala Limestone, while the majority (24) were located in the Avon Park Formation and Oldsmar Formation [130]. However, some non-organic clustered high values of Hg were measured in dolostones at various depths (Figure 3). There is clear evidence that atmospheric deposition of Hg has been occurring in southern Florida for at least the last 56 MY (Avon Park Formation in Table 4 and data from the Lower Eocene section in Figure 3). Note that these measurements are substantially higher than those made by the Florida Geological Survey (Table 4; μg/g is 1000 times larger than μg/kg). The high degree of instrument calibration for these data [130] suggests they may be more reliable measurements than the previously published data (Table 4).

### 4.2. Atmospheric Deposition of Hg in the Southern Florida Environment

The primary natural source of Hg deposition in southern Florida is from the atmosphere [91,132]. However, herein lies a paradox concerning what is defined as natural and what is anthropogenic. The most recent investigations of Holocene atmosphere mercury concentrations in comparison to the peak concentration caused by anthropogenic impacts is a ratio ranging from 3 to 14.4, with the best estimate of Holocene atmospheric Hg concentration being 0.27 ± 0.11 ng m^−3^ [72]. The associated deposition rate for the Holocene (pre-man impacts) is 1.5 ± 1.0 μg m^−2^ y^−1^ (Table 1). This natural deposition rate only increased by 0.2 ng m^−3^ during the period 1500 to 500 CE; therefore, anthropogenic impacts on atmospheric deposition became significant after 500 CE.

Hg accumulation in the southern Florida environment is mostly concentrated in organic soils, as demonstrated over the past 56 MY based on the stratigraphic distribution of Hg in the carbonate rocks underlying the region (Figure 3 and Figure 4). This implies that organic soils associated with wetland environments are expected to have the highest concentrations of Hg [133]. Therefore, it is logical that the peatlands of the Everglades, which is the largest wetland area in southern Florida, is the largest reservoir of accumulated Hg [92,134,135,136,137]. There is a general paucity of Hg data on other wetland soil deposits in southern Florida for comparison to the Everglades Hg database [138].

The historical area of the Everglades peatlands was approximately 7000 km^2^ [139], and the deposition time ranged from 3000 to 8000 years covering the total peatland area depending on the location, original thickness of peat, and the accuracy of the age determinations [140,141,142,143,144]. Therefore, Hg deposited before any significant global anthropogenic impacts occurred from between 2500 and 7500 years based only on the global changes, as shown in Table 1. However, localized impacts to atmospheric Hg concentrations only began to become significant perhaps conservatively at 1900 AD, leaving a range of “natural” deposition from 2880 to 7880 y. Therefore, “natural” atmospheric Hg deposition in the Everglades has occurred for approximately 4000 y (average timeframe before significant anthropogenic impacts and considering the entire area of the Everglades).

If the Enrico et al. [72] Holocene total Hg deposition rate of 1.5 μg m^−2^ y^−1^ is used as a proxy, the total pre-anthropogenic deposition for the 7000 km^2^ peatlands area would be approximately 42,000 kg (42 metric tons). If the average Hg deposition rate was 25 μg m^−2^ y^−1^ during the past 120 years (Table 1), the total amount of anthropogenic mercury deposition would be 280 kg or 150 times less that of the “natural” deposition quantity. Therefore, an estimated reservoir of mercury in the Everglades peats is approximately 42.3 metric tons. This method of estimating the atmospheric deposition of Hg into the Everglades peatlands cannot be considered to be accurate based on the unknown past concentrations of local Hg in the aerosol fallout, but does give a general order of magnitude estimate. This is an extremely large reservoir of legacy Hg.

**Figure 5:**
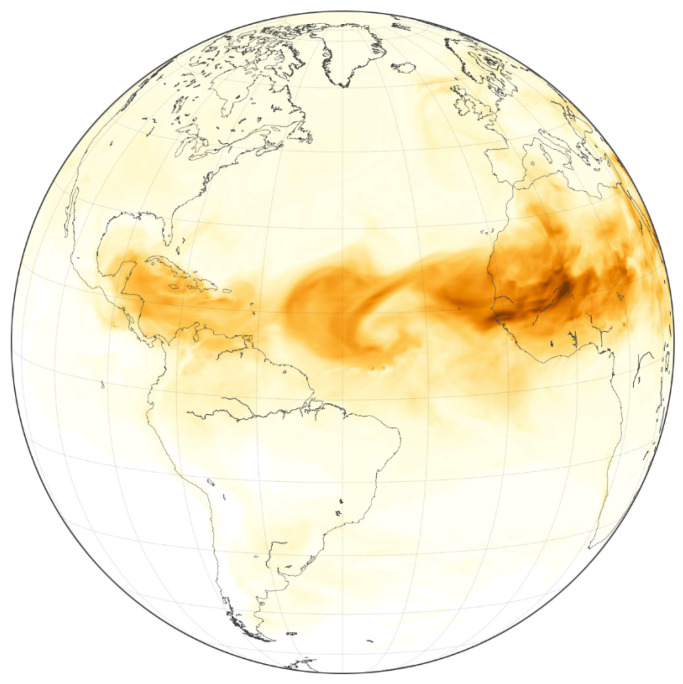
Image acquired by NASA GOES-5 Satellite of Saharan dust plume extending from Africa across the Atlantic Ocean on 28 June 2018 (NASA online) [150].

Hg was and is transported to southern Florida within Saharan dust from Africa (Figure 5), but few data have been collected to ascertain the concentrations and the form. Mason et al. [35] measured the Hg content in particles collected over the North Atlantic that are assumed to be from Saharan dust. Streets et al. [145,146] estimate that ASGM dust emissions from the Sahara and sub-Sahara area range between 0.154 and 0.300 kt/yr. Hg in the Saharan dust may be attached to clay minerals. The estimated Saharan dust emission to the atmosphere, accounting for seasonal and annual fluctuations, ranges from 0.4 to 2.0 billion t/yr [147].

Studies of atmospheric Hg occurrence by the University of Arizona and the U.S. Geological Survey have found that in the smallest aerosol particles (approximately 1 micrometer) in African dust, Hg occurs at a concentration of up to 2 μg/g [148]. The source of this mercury is believed to be open-pit mines in Algeria and the rock formation from which the Hg is mined [148]. Some Hg analyses have been performed on Saharan dust samples collected at Cabo Verde in the Canary Islands. Bailey [149] reported that the Cabo Verde dust sample had a mean Hg concentration of 49 ± 31.9 ng/g and a corresponding atmospheric concentration of 2.7 ± 2.37 ng m^−3^. Two additional samples of Saharan dust were collected at this location and yielded Hg concentrations of 37.5 and 51.8 ng/g. The Cabo Verde data are useful but may be enriched by local Hg sources in volcanic debris that is common in that area.

The rate of African dust deposition in North America may slow based on future global warming. Some modeling of various wind impact scenarios applied to the African land surface show lower rates of entrainment [151]. However, the warming surface of the ocean near land offshore from Florida may increase the discharge of volatile Hg dissolved in the seawater. The Atlantic Ocean produces an annual flux of gaseous Hg of approximately 840 Mg/yr [152]. Therefore, the future rate of onshore movement of Hg from African dust and marine aerosols is an open issue with consideration to the southern Florida environment.

### 4.3. Naturally Occurring Hg Sources in Southern Florida Soils, Wetland, Lake and Tidal Sediments

An investigation of Florida’s soils to establish baseline concentrations of 15 trace elements was conducted by Chen et al. [153]. In the 443 samples analyzed for their Hg concentration, the range in concentration was 0.2–430 μg/kg, with a median value of 4.31 μg/kg and a mean of 4.2 μg/kg. The baseline concentration range was 0.75–39.6 μg/kg compared to the range for the entire USA of 9.1–368 μg/kg. Note that the large range in values with some very high concentrations was caused by the sampling of some environments in which higher concentrations of all metals were found (e.g., accumulated urban runoff or agricultural sites with past pesticide use). Chen et al. [153] determined that the strongest relationship between Hg and other metals was for Fe and Al. Bacause of anthropogenic source of Hg in the Florida environment, the baseline upper values are likely not to be natural. However, the correlation of iron and aluminum suggests deposition from Saharan dust, based on general elemental enrichment linked to the composition of particulate matter in southern Florida [154]. Therefore, the natural concentration should be within the baseline range.

**Figure 6:**
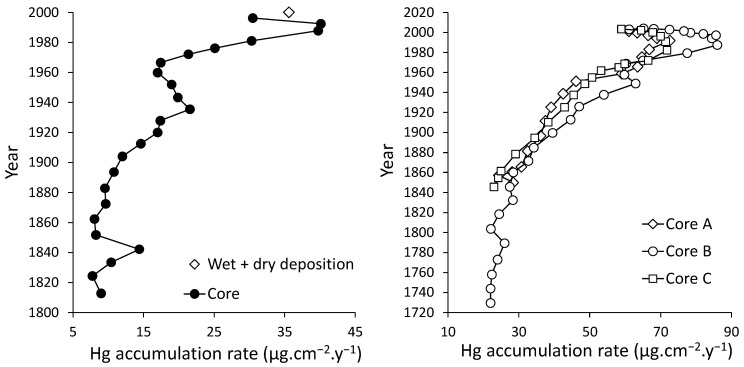
Hg accumulation from two cores collected and analyzed in southern Florida. The data on the left are from site 3 in Water Conservation Area 2A (from Abelak et al. and obtained by T. Atkeson) and the data on the right are compiled from 3 cores from the western edge of Lake Annie as analyzed by Engstrom et al. [155]. (Original government report as modified from Pollman and Engstrom [92]).

The Chen et al. [153] dataset does suggest that an association occurs between the concentration of organic carbon in the soil and the higher Hg concentrations found in the peatlands of the Everglades. Two sediment cores were collected for Hg analysis, one from a location in Water Conservation Area 2A (WCA) in the Everglades (from T. Atkeson as reported by Pollman and Engstrom [92]), and one from Lake Annie, located approximately 40 km northwest of the western edge of Lake Okeechobee [155]. The WCA core suggests that the pre-industrial Hg deposition rate in southern Florida could be as low as 10 μg/m^2^-yr, but the Lake Annie core suggests it being slightly over 20 μg/m^2^-yr (Figure 6). In both cores, the rate of mercury deposition began to increase significantly in approximately 1850 and peaked between 1985 and 1990 at approximately 40 μg/m^2^/yr in the WCA core and at 84 μg/m^2^/yr in the Lake Annie core. The WCA core generally follows the values in the European bog as reported by Enrico et al. [72], but the Lake Annie core has a pre-industrial baseline concentration more than double of that measured by Enrico et al. [72]. The peak values of Hg in 1990 were also more than double of those found in Europe by Enrico et al. [72]. If the Lake Annie values would be correct, the pre-industrial Hg deposition in the Everglades could be double of that estimated using the Enrico et al. [72] dataset, or over 84 kg. Pollman and Engstrom [91] suggest that the Lake Annie cores were impacted by “sediment focusing” which caused a preferential deposition pattern of fine sediments moving into the deeper part of the lake, thereby increasing the concentrations of Hg. When corrected to data from Lamborg et al. [156], the values were closer to the WCA core data. The WCA core seems to indicate that the lower value has a higher likelihood of being a more accurate estimate. There were a few other cores collected in southern Florida, but they did not have a favorable sedimentological regime and are not considered to be as accurate as the WCA and Lake Annie cores [92].

In Section 4.2, a proxy for the natural rate of atmospheric Hg deposition in southern Florida of 1.5 μg m^−2^ y^−1^ was used to estimate the natural amount of Hg occurring in the Everglades peatlands before modern alteration. The WCA core pre-industrial value of 10 μg m^−2^ y^−1^ suggests that the estimate could be 90% higher. The higher influx of Hg to southern Florida could, in fact, be true based on the impacts of Saharan dust and ocean flux, whereas these factors do not impact the two Pyrenean peat bogs where the estimates for a “natural” condition were made by Enrico et al. [72]. If the higher natural influx rate of 10 μg m^−2^ y^−1^ is used, then the Everglades received approximately 280,000 kg (280 metric tons) of Hg. The historic influx at a higher concentration of Hg would not be significant in this case. Therefore, the range of mass of Hg in the Everglades peatland lies between 42.3 and 280 metric tons.

No high-quality Hg datasets could be found for undisturbed natural wetland areas in freshwater environments of South Florida, such as cypress domes, cypress sloughs, or other isolated wetland types. Stratigraphic samples need to be collected and analyzed from wetland sites that are undisturbed and remote from the Everglades. However, very high Hg concentrations (methylmercury) were found in a constructed wetland area, as documented by Rumbold and Fink [138].

Hg has been measured within four cores collected in the southern Everglades and Florida Bay [157]. Only one of these cores (Coot Bay) penetrated deep enough to reach pre-industrial-age sediments. In this case, the measured Hg flux rate was between 20 and 30 μg m^−2^ y^−1^, which is two to three times higher than that found in the WCA core. It is not possible to assess the relationship between the freshwater and marine Hg data. Kang et al. [157] suggested that anthropogenic enrichment of Hg may be the result of the chemistry of runoff entering the natural system. However, in another investigation, Rumbold et al. [158] concluded that atmospheric deposition was the most important factor causing Hg enrichment in the sediments and runoff was not a significant factor.

Atmospheric concentrations of particulate Hg in air samples collected at the shoreline in Broward County averaged 34 pg m^−3^ [159]. The likely source for this Hg is Saharan dust. Shinn [148] suggests that the dust could contain up to 2 μg/g of Hg, which is much higher that the measured concentrations at the shoreline. If the natural flux of atmospheric Hg deposition of 10 μg m^−1^ y^−1^ is assumed to be a proxy for all of southern Florida, including Monroe, Dade, Broward, Palm Beach, Hendry, Collier, and Lee counties, the annual Hg flux would be 257.1 kg/y. If the current Hg atmospheric flux is greater than the prehistoric value, the overall deposition rate in southern Florida could be as great as double the value, or 514.2 kg/y.

### 4.4. Oceanic Evasion of Hg into the Southern Florida Environment

Hg that occurs within oceanic seawater is recycled back into the atmosphere by surface evasion processes. When evasion occurs in nearshore waters, the prevailing wind can move the Hg inland and it becomes another source within the Hg budget.

Dvonch et al. [159] conducted an investigation of Hg measured as vapor phase, particle phase, and in-event precipitation. Their site 1 location was less than 100 m from the beach, and they assumed it was the baseline for Hg moving from offshore to onshore with consideration of the wind direction. The vapor concentration was 1.8 ng/m^3^ at the background station. The particle concentration at the shoreline site was 34 ng/m^3^, which was 50% lower than the inland site measurements. While they could not determine the absolute source of the Hg, there is a high likelihood that the vapor Hg at the shoreline is the combination of marine evasion and the vapor phase of the atmosphere. The particle phase Hg may be a proxy for Saharan dust transport at the shoreline, based on the assumption that no upwind industrial or anthropogenic Hg sources exist.

Sunderland and Mason [160] and Mason [34] estimated that the evasion rate for the North Atlantic Ocean is 0.15 to 19 ± 1.3 nmol/m^2^/day. Therefore, the onshore wind in southern Florida does carry evasion-source Hg onshore at significant concentrations. However, because of water temperature variability, which controls the evasion rate, and wind direction changes, it is not possible to accurately estimate this source of Hg to southern Florida. However, based on some calculations of averages, the range of values should lie somewhere between 2 and 4 kg/yr.

## 5. Anthropogenic Sources of Hg in the Southern Florida Environment

### 5.1. Anthropogenic Hg Deposition in Southern Florida: Sources, Accumulation, and Recycling

Nearly all the fluxes of Hg into the southern Florida environment are now anthropogenic or the result of recycling because even the atmospheric concentration is enriched compared to the pre-industrial era and has not declined back to historic background concentrations [33,92]. In fact, global Hg emissions have risen slightly between 2010 and 2015 [145,146]. Therefore, the Hg budget for southern Florida contains the gross evasion Hg from the Atlantic Ocean [34,152,160,161], which becomes part of the wet and dry atmospheric fallout, and moves onshore via the wind. The in situ atmospheric flux comes from power plants, incinerators, and cement plant discharges, along with gaseous emissions from wastewater treatment facilities. Wildfire, muck fires, and sugar cane fires tend to remobilize Hg from the soil and plant storage back into the atmosphere. Discharges to the soil and aquifer system occurred through past practices, which included the use of pesticides containing Hg, unlined landfill leachate discharge, illegal dumping of materials containing Hg, and discharges from large reservoirs of Hg, such as the Everglades peatland area. The influx of Hg into southern Florida is greater than the outflow with the tendency for Hg accumulation in wetland soils, the largest being the Everglades peatlands, natural and man-made lake and pond sediments, estuary sediments, within vegetation (both in the wood, stems, and leave material), and through accumulation in periphyton mats, fishes, and macrophytes.

**Figure 7:**
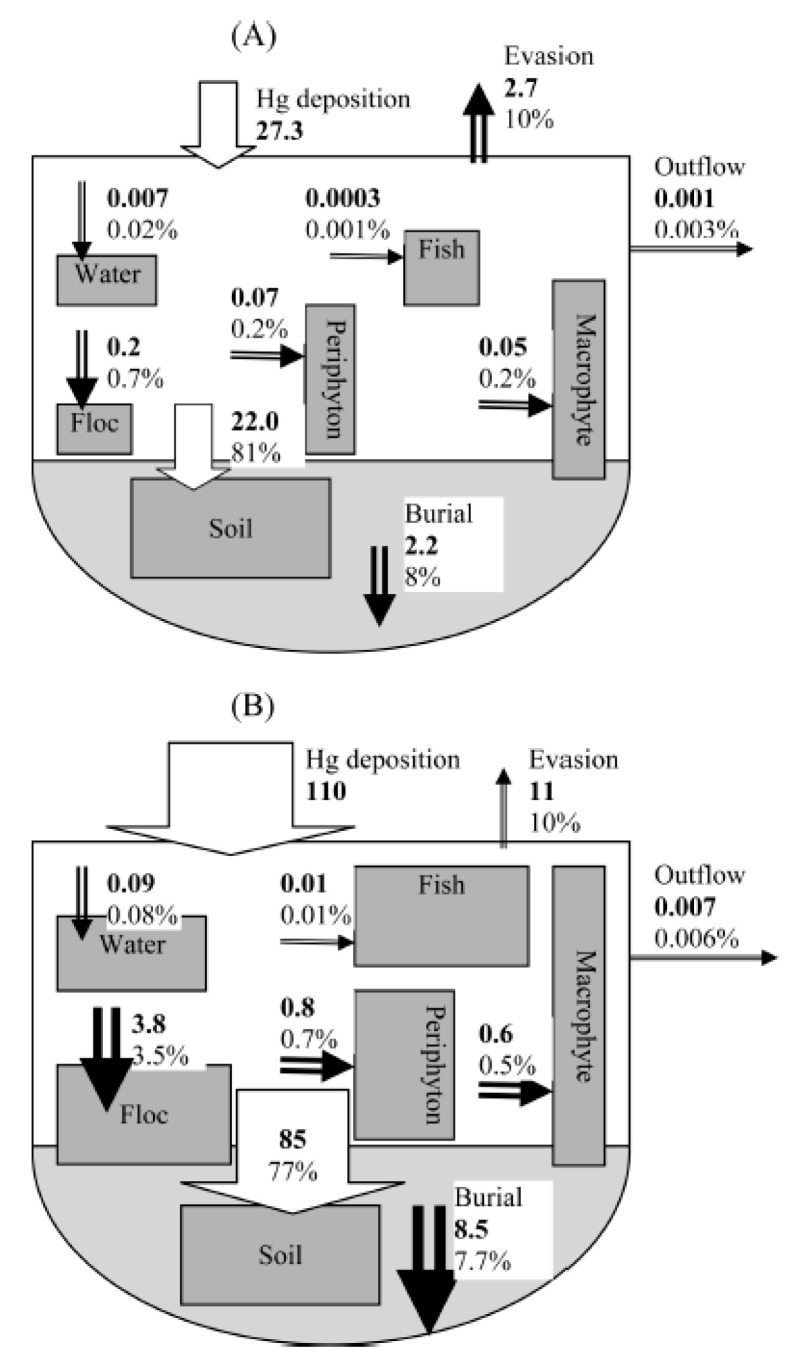
Estimated mass (bold, in kg) and fraction (%) budget of total Hg to the Everglades in the dry (**A**) and wet (**B**) seasons for 2005 (from Liu et al. [162]). The size of the arrows and boxes show the relative masses for seasonal comparison.

Perhaps a good illustration of the Hg balance or budget in southern Florida is an example from the Everglades (Figure 7). Liu et al. [162] developed a Hg mass budget for the Everglades as a whole, which can be applied to other site-specific locations in south Florida where some local fluxes could be lower or higher within the budget.

A very important factor in Hg recycling within the Everglades is the evasion of Hg, constituting only 10%, and 0.001–0.007% of the deposition. This loss likely occurs via diffusion from surface water bodies and perhaps some via transpiration from plant leaves. There is another critically important factor missing from this mass budget, which is the loss of soil via oxidation and muck fire volatilization with subsequent down-wind removal (arrows added). In some locations there has been a loss of several meters of peat due to oxidation [142,163]. The estimated volume of peat loss is 4 × 10^9^ m^3^ [163]. Whether this mass of volatile and mobilized mercury stayed within the Everglades system or was discharged west or northwest away from the Everglades, based on the predominant wind direction, is unknown, but an important issue. This same type of mass budget could be developed for other areas from small-scale wetlands to a full ecosystem. This is a critical issue impacting plant communities, wildlife, fish, and direct (soil ingestion or contact and breathing in Mg) or indirect (ingestion of contaminated food) human health in southern Florida.

### 5.2. Historical Use of Hg Compounds in Agriculture and Common Domestic and Industrial Products in Southern Florida

Hg has been used as a preservative in products sold in southern Florida and as a pesticide from the late 1890s to the mid-1960s. A series of hydroxymercurichlorophenol substances were used as insecticides on some vegetable and field crops [80]. The earlier use of these compounds on sugarcane was cancelled in the 1960s. Therefore, legacy Hg is contained within permitted and unpermitted landfills, soils, freshwater body sediments, estuarine sediments, and tidal water body sediments in southern Florida.

One common Hg use was the preservation of gladiolus bulbs to prevent mold formation using the compound mercuric arsenate. Several hazardous waste sites in Lee County, Florida contained this substance near former bulb harvesting facilities. During interviews (for an environmental site assessment) of a former worker that engaged in soaking the bulbs at a gladiolus production facility located in northwest Lee County, it was discovered that of the 42 employees that worked at the site for more than 5 years, all had died of cancer (100%) within 20 years after their employment. Cyano (methylmercuric) guanidine, mercurous chloride, N-Methylmercuri-1,2,3,6-tetrahydro-3, 6-endomethano-3,4,5,6,7,7-tetrachlorophthalimide, phenylmercuric acetate, phenylmerxcuric trithanol ammonium lactate, and sodium methylmercury thiosalicylate were also used to soak gladiolus bulbs [80]. Cuttings from the gladiolus plants were commonly treated with cyano (methymercuric) guanidine or methymercury hydroxide prior to shipment. Various Hg compounds were also used on flowering and foliage plants as a foliar treatment before shipment. Hydroxymercury chlorophenol was commonly used in southern Florida in the treatment of sacks (burlap) containing seeds, and the seeds used to raise crops [80]. Ornamental flower seeds were treated with various Hg-containing substances. Various trees and shrubs have received soil disinfection with Hg compounds, while the direct treatment of wounds or for disease protection were other uses, but these cannot be documented in southern Florida.

Golf course turf grass was widely treated in the past using fungicides containing Hg [80,164]. While recent research has focused on the use of arsenic-containing compounds on golf courses, the past use of Hg compounds has resulted in the enrichment of biota in golf course ponds and wetlands [165]. At one time, Hg-containing substances were used in turf weed control, in particular phenylmercuric acetate [80]. While South Florida contains hundreds of golf courses, only the ones over 30 years in age are likely to contain residues of the Hg applied as a fungicide.

The domestic and industrial use of Hg compounds was very common until strict legislation and environmental rules were enacted to ban its use. Some of the most common uses are listed in Table 5. Disposal of the waste products from these materials is another reason why old domestic solid waste and agricultural landfills are so enriched in Hg.

Various Hg compounds were used in various ointments applied directly to human skin, such as metallic Hg and ammoniated Hg for louse control [80]. Bathing caused Hg to be cleaned from the skin and enriched domestic wastewater with Hg, creating a pathway to streams and groundwater (septic tanks).

High concentrations of Hg occur in dental amalgams based on the phase of Ag_2_Hg_3_ (18.9 ± 1.5%) used or another compound such as (Hg, Cu)-S (7.1 ± 1.7%) [166]. This Hg occurrence can impact emissions from biomedical incinerators or crematory facilities, and soils and groundwater in the vicinity of cemeteries.

### 5.3. Atmospheric Emissions and Deposition of Hg in Southern Florida from Power Plants, Other Large Emission Sites, and Sources

**Figure 8:**
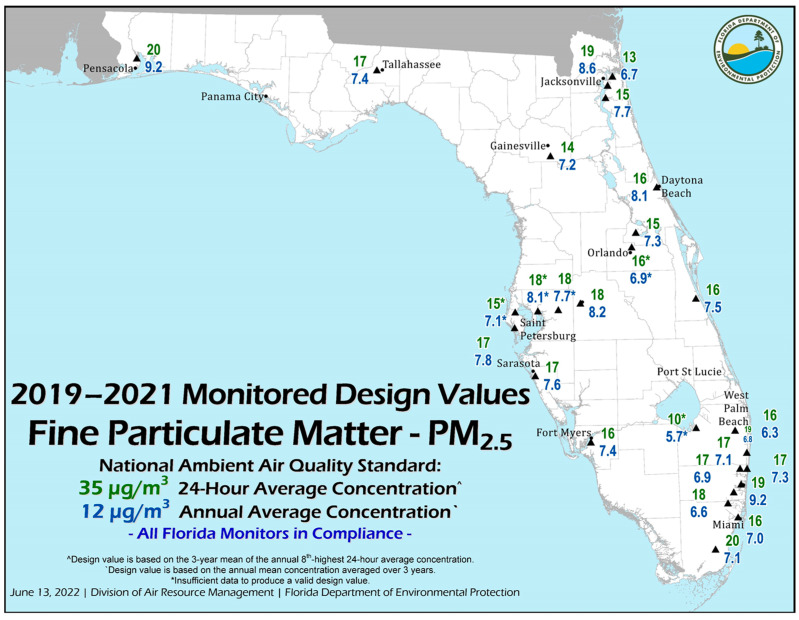
Major air emission facilities in southern Florida permitted by the Florida Department of Environmental Protection, including power plants, industrial discharges, solid waste incinerators, and cement kilns. Map from the Florida Department of Environmental Protection.

Based on data obtained from the Florida Department of Environmental Protection, there are 11 large air discharge permits issued in southern Florida (Figure 8). The permitted values for particulate matter (PM_2.5_) for the 24 h and annual average concentrations are shown in the figure. Of the 11 facilities, 9 occur in the southeast coastal area, with one in the south-central area near Belle Glade and one in the southwest area near Fort Myers. Many of these facilities have some level of atmospheric Hg discharge. Most of the large air quality permits are associated with power generation and two for cement plants.

**Figure 9:**
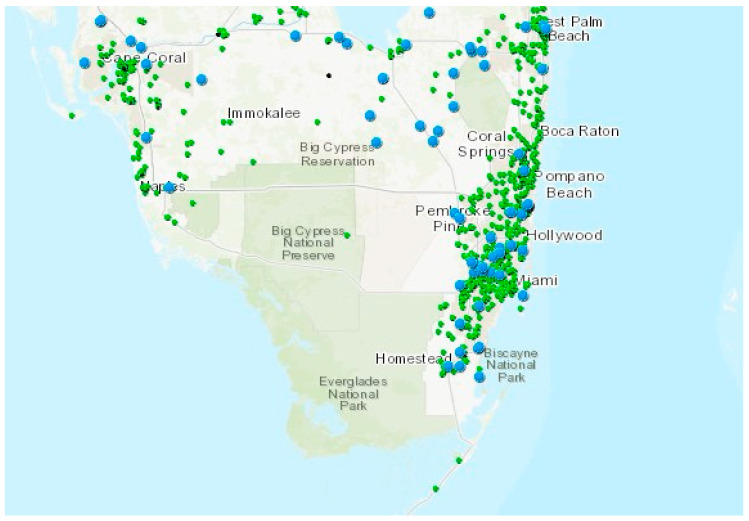
Nearly all facilities with air emissions permits in southern Florida, including power plants, and small and large industrial facilities which include animal and human crematories, asphalt plants, bulk gasoline plants, cement batching plants, resource recovery and reclamation facilities, printing operations, and others. Only a few of these facilities have actual Hg emissions. Map from the Florida Department of Environmental Protection.

A map showing most of the current air quality permits issued in southern Florida is shown in Figure 9. Many of these air discharge permits contain little or no Hg flux into the atmosphere. Some asphalt plants are also included in the permits. These facilities can discharge small quantities of Hg because the oil-based product contains some Hg, as found in emissions from oil-fired plants. The concentration of GeM in asphalt is approximately 0.27 ng/g [167]. The density of the permits is directly linked to large population centers, with the greatest number of facilities occurring in southeast coastal region.

Approximately 50% of atmospheric Hg emissions in the United States originate in coal-fired power plants [168]. Gaseous oxidized Hg from large power plants commonly occurs as dry deposition and tended to decrease after these generation facilities invoked mandated Hg discharge reductions [58]. Hg emissions were cut by nearly 90% with the implementation of the Mercury and Air Toxics Standards rule [168]. Fortunately, southern Florida no longer contains any operational coal-fired power plants. However, oil-fired and gas-fired power plants, solid waste incinerators, and cement kilns are still operating. There are few studies of Hg discharges from southern Florida facilities, but annual stack emissions tests are required for most facilities that have air quality permits issued by the Florida Department of Environmental Protection.

**Figure 10:**
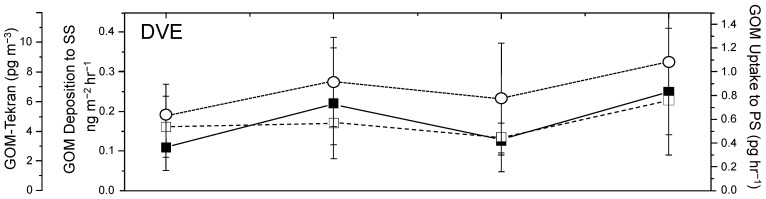
Seasonal means +/− of Tekan-GOM air concentrations (GOMT). GOM dry deposition to a surrogate surface (GOMSS), and GOM uptake to a passive sampler (GOMPS) measured at station DVE located at Fort Lauderdale, Florida (modified from Gustin et al. [9]). The symbols used are is the order of the Hg types mentioned in the caption.

Gustin et al. [9] conducted a study of Hg dry deposition at three sites including the Port Everglades/Fort Lauderdale area in southeast Florida. The deposition quantity of GOM varied between winter, spring, summer, and fall, with the highest rates occurring in the fall (Figure 10). Five point-source discharge facilities were located in the area, including three domestic waste to energy incinerator plants (Wheelabrator North, Wheelabrator South, and Covanta), a natural gas-fired power plant (Fort Lauderdale), and an oil-fired power plant (Port Everglades). The annual rates of mercury discharge (Total Hg, GOM, and PBM) in 2002 for the five sites in kg/y were 46, 26, and 9, 53, 31, and 11, 7, 4, and 1, not recorded, and 13, 4, and 3, respectively. The predominant wind direction during most of the year at this site is from the east or southeast. In the summer months, the sea breeze is the dominant factor. Winter frontal passage tends to provide some strong winds from the northwest and northeast. The historical operation of power plants has contributed to the accumulation of Hg in the southern Florida environment but is not a significant factor in the legacy Hg in the Everglades.

The last coal-fired power plant in southeast Florida (Martin County) closed on 1 January 2021 and is being replaced by solar field facilities (from Florida Power & Light Company, Juno Beach, FL, USA). There are currently five gas-fired power plants operating in southern Florida. The facilities and estimated annual Hg emissions are as follows: Fort Myers (0.000002 tons in 2022, below threshold for past 3 years), West County, Palm Beach County (below threshold 2022–2015, 0.000230 tons in 2016, 0.000159 tons in 2017), Fort Lauderdale (below threshold 2017–2022, 0.000001 tons in 2015 and 0.000014 tons in 2016), Port Everglades (no H-114 emissions reported), and Turkey Point (no H-114 emissions reported for small gas-fired plant). The cumulative emissions from these plants on an annual basis are not significant. Nine waste incinerators are operated in southern Florida, and include the following: Broward, Wheelabrator South Broward (72.1 kg/yr); Hendry County, U.S. Sugar (49.9 kg/yr); Lee County (52.6 kg/yr); Lee County East Water Reclamation (35.8 kg/yr); Miami, Dade County (32.7 kg/yr); Palm Beach, County-#1 and #2 (46.7 kg/yr); Palm Beach County, Glades Sugar House (33.6 kg/yr); Palm Beach County, Okeelanta Cogeneration Plant (21.3 kg/yr); and Palm Beach County, Osceola Farms (19.1 kg/yr). The estimated emissions of Hg from 2011 to 2020 in kg/yr is in brackets after each facility’s name (from the Florida Department of Environmental Protection based on 2011 to 2020 data). The waste to energy incinerators emitted approximately 204.1 kg/yr and the other incinerators emitted approximately 159.7 kg/yr.

### 5.4. Atmospheric Emissions of Hg from Cement Plants

In China, the cement industry has become one of the largest emission sources for Hg [169,170], and is a source of mercury discharge in the United States [171,172,173]. The source of the Hg is not primarily from the limestone treated in the kiln, but from the fuel used to fire the kiln. There are two cement production facilities located in southern Florida run by Cemex and Titan America. The estimated annual emissions of Hg for these two facilities are 54.9 and 95.7 kg/yr, respectively [172]. Therefore, the atmospheric flux of Hg from cement plants into the southern Florida atmosphere is approximately 150.6 kg/yr.

### 5.5. Hg in Soil Amendments (e.g., Wastewater Sludge) and Domestic Wastewater Used for Irrigation

Hg commonly occurs in nearly all municipal wastewater treatment systems, with concentrations dependent on the percentage of industrial wastewater entering the system and the degree of pretreatment before entering the municipal system [174,175,176]. If the wastewater sludge is burned, Hg control is required within the system [177]. Soils impacted by wastewater treatment discharges and sludge can be enriched in methylmercury depending on the composition of the sludge and the amount of iron in the sludge [178]. Carpi et al. [54] documented the release of Hg into the atmosphere from soils amended with wastewater sludge. In 2021, the Hg concentration in wastewater sludge for the southwest Florida City of Cape Coral was reported to be 0.29 mg/kg (personal communication from Jeff Pearson, City Utility Director). Data on mercury concentrations in wastewater from other facilities were difficult to obtain. There are no published compilations.

In southern Florida, highly treated wastewater is commonly sprayed on fields for disposal and used for irrigation on residential and various other municipal properties, such as common landscapes, parks, and school facilities [179,180]. The City of Cape Coral uses 100% of its domestic wastewater discharge in the dry season for irrigation [181]. Typical Hg concentrations in the treated domestic wastewater effluent are low at 0.031 mg/L (personal communication from Jeff Pearson, City Utility Director). Many facilities report Hg concentrations below detection limits.

### 5.6. Industrial and Landfill Aerosol and Groundwater Discharges

Groundwater can become contaminated with various chemicals containing Hg, particularly from sites where waste materials are concentrated. Nevondo et al. [182] documented the Hg concentrations in soils and groundwater discharge (leachate) from domestic landfills. The source of the Hg was primarily from domestic waste materials containing Hg, such as florescent light blues, batteries, electric switches, thermometers, and other materials [183]. Lindberg et al. [54] documented the present concentration of Hg in landfill leachate and in internal gas. Monitoring of gas discharges at the Brevard County landfill showed concentrations of total gaseous Hg ranging between 6600 and 7700 ng/m^3^. The Hg in leachate for 14 Florida sites showed measurable concentrations of Hg but there were no reports of meaningful concentration values [184]. Reinhart and Grosh [185] tested landfill leachate for the presence of Hg at 39 sites throughout Florida and found a mean concentration of 0.696 μg/L.

Atmospheric discharges of Hg were also present through the clay cap and the landfill gas collection vents in Martin County and Palm Beach County sites [50]. The annual estimated flux of Hg into the environment for the Martin County and Palm Beach County landfills was 80–100 and 20 g/yr, respectively. The estimated total Hg discharge from all class I landfills in Florida was estimated to be 10 kg/yr and in southern Florida is approximately 4 kg/yr, which is substantially less than other major sources of atmospheric emissions including power plants and waste incinerators.

### 5.7. Atmospheric Aerosol Emissions Deposition from Muck and Forest Fires

The average annual global emission of Hg caused by biomass burning for the period 1997–2006 is 674 ± 249 Mg^−1^ [186]. This constitutes approximately 8% of the combined natural and anthropogenic emissions. The issue of Hg emissions caused by biomass fire mobilization is particularly relevant to southern Florida because of two issues: first, a large portion of the deposition of Hg originates from the onshore movement of atmospheric aerosols incorporated in Saharan dust, and secondly, the remobilization of Hg from vegetation and from peat is a major process of concern.

Particulate Hg is part of the Saharan dust that is deposited in southern Florida, but is likely a small portion, whereas the overall atmospheric fallout is likely GEM associated with the atmospheric circulation pattern that transports the dust [148]. African savanna grasses have Hg concentrations ranging from 6 to 9 μg/kg [186]. Fire mobilization from African vegetation surely adds to the westward-bound atmospheric aerosols that cross the Atlantic Ocean. In southern Florida, Greenplate et al. [187] measured mercury in willow tree trunks with a mean value of 11.91 μg/kg and a mean of 4.26 μg/kg in the leaves. Approximately 90% of the mercury in the leaves was deposited on the leaf surface. It should be noted that the sampling locations in southwestern Florida are downwind of large legacy mercury deposits (e.g., the Everglades peatlands). Fortunately, willow is a wetland plant and does not commonly burn; therefore, it returns some Hg back to the environment via litter fall with a contribution to the soil but has a minimal atmospheric contribution.

A study of the dynamics of Hg uptake and movement within sawgrass (*Cladium jamaicense*) was undertaken by Meng et al. [188]. They used a multi-isotope technique to measure the uptake of Hg into the roots and translocation into the leaves. Significant quantities of mercury moved from the soil into the roots and upward into the leaves. The mercury did not exit through the leaves into the atmosphere. This is an important issue with regard to the Hg dynamics of the Everglades peatland Hg reservoir because the Hg trapped in the soils is transported to the leaves within the concentration in the leaf mass and is based on the plant age and the concentration of Hg in the underlying soil. The storage of Hg in the sawgrass leaves exposes the Hg to fire, which then conveys the Hg back into the atmosphere, where it moves downwind until it is deposited as wet or dry fallout or by absorption or adsorption to plants’ leaves at a new location. This mechanism for wildfire Hg mobilization does not necessitate muck fires to recycle legacy peak into the atmosphere and, therefore, affects natural and/or management areas of the Everglades.

Another potential source of remobilization of Hg from the Everglades peatland soils to the atmosphere is via controlled burning during sugarcane harvesting. No data in southern Florida could be found on the metal concentrations in sugarcane plants, but a study conducted in Australia found Hg concentrations ranging from 0.02 to 0.20 mg/kg, based on only six analyses [189]. If similar concentrations are found in southern Florida sugarcane, then the Hg mobilization from burning of the plant leaves would be low. However, the impact on oxidation of the upper part of the peat could create a greater degree of mobilization.

### 5.8. Hg Emissions from Vehicle Exhaust

Hg occurs as a trace element in unrefined petroleum as in all naturally occurring organics in rocks, sediments, condensate, and natural gas [190,191,192]. The mean concentration of all forms of Hg in 170 oil streams in the United States in 2004 was 3.5 ± 0.6 μg/kg [192]. The measured concentrations of Hg in common refined fuels are 571.1 ± 4.5 ng/L for gasoline, 185.7 ± 2.6 ng/L for diesel, and 123.3 ± 23.5 ng/L for liquefied natural gas (LPG) [193]. Therefore, trace amounts of Hg occur in all refined petroleum products. Hg is, therefore, released to the atmosphere beginning at the refining stage [194]. In addition, Hg discharge occurs during burning to produce electricity and when burning as fuel in vehicles and during other uses (e.g., home heating, industrial uses, etc.).

While the Hg emissions from the large-scale use of petroleum products for power generation and heating in the cement-making process are the largest point sources of atmospheric Hg discharge in southern Florida, the discharge of Hg from vehicles is also important. In a controlled test, Won et al. [193] measured the concentration of Hg in the exhaust of vehicles idling and at driving speeds using different fuels. The results of these tests showed that for idling the Hg emissions concentrations for gasoline, diesel, and LPG were 1.5–9.1, 1.6–3.5, and 10.2–18.6 ng m^−3^, respectively. In the driving mode, the emissions for gasoline, diesel, and LPG were 3.8–16.8, 2.8–8.5, and 20.0–26.9 ngm^−3^, respectively. While these values are significant, even electric vehicles have indirect emissions of Hg into the atmospheric because the recharge requires electric power generation [195]. Where the power generation is coal-fired, the Hg emissions for electric vehicles can be 92% higher than using gasoline in the vehicles as fuel [195], but in southern Florida electric power generation is a blend of nuclear, solar, and combined-cycle natural gas, which lessens the impact of vehicle operation on atmospheric Hg discharges.

The total Hg emissions for southern Florida can be estimated using the number of registered vehicles in Florida and estimating the number in southern Florida that are operating and an estimate of the total volume of gasoline and other fuels consumed. Based on compiled statistics from the Florida Department of Transportation, there was an average of 17.6 million vehicles registered in Florida over the past 10 years. A study determined that light-duty, gasoline-powered vehicles discharge 0.31–1.4 ng/mi of Hg [196]. Diesel-powered vehicles discharge 6.3 to 11.0 ng/mi [196].

Approximately half of the registered vehicles operated at least part of the time in southern Florida. In Florida, 193,841 barrels of gasoline were consumed in 2020. Therefore, 8.1 million gallons of gasoline were consumed, with perhaps 40% of this consumption occurring in southern Florida. A typical concentration of Hg in refined gasoline is 571 ± 4.5 ng L^−1^ [193]. Therefore, a barrel of gasoline contains 158.9873 L and contains approximately 90,782 ng of mercury. If it is assumed that 100% of the Hg in the gasoline is discharged into the atmosphere, approximately 1114 g (1.1 kg) of Hg was discharged in the southern Florida atmosphere in 2020, which is small compared to the incinerator, cement plant, and power plant discharges. A calculation of diesel-drive discharges is approximately 0.3 kg of mercury in 2020.

### 5.9. Hg in Urban Stormwater Management Facility Sediments and Street Sweepings

As stormwater carries pollutants, it is regulated under the U.S. 1977 Clean Water Act under the 1972 National Pollutant Discharge Elimination System (NPDES, EPA). One method to capture this pollution coming from the drainage of various catchment areas consists of diverting urban runoff into receiving bodies which act as decantation pits, as well as encouraging the phytoremediation of nutrients in particular. In southern Florida, dry but mostly wet detention/retention areas are used to capture runoff and thus not only attempt to mitigate flooding [197], but especially capture pollutants in the dissolved and particulate forms. Statewide stormwater regulation in Florida was established by the Environmental Regulation Commission in 1982 (ERC; Chapter 17–25 F.A.C), following the earlier establishment of interim guidelines in Chapter 17–4.248, F.A.C., revised in 1982 as rule Chapter 17–25 F.A.C. The formal rule specifies, e.g., the use of wet detention ponds (i.e., permanent water bodies dug below the water table, and which would discharge, at a certain elevation threshold, to receiving hydrosystems) as manmade structures to be built alongside the construction of residential and commercial developments. The Water Resource Implementation Rule (WRIR) established in 1990 further sets performance standards with an 80% reduction in annual average loading of total suspended solids for most discharges besides those discharging into Outstanding Florida Waters (OFWs), for which the retention is set to 95%. Standards for the reduction of total nitrogen (37–60%), total phosphorus (TP, 59–85%), and metals (40–80%) have also been implemented. Even though it is estimated that there are over 70,000 urban stormwater ponds in Florida, little is known about their sediment accumulation rate and characteristics [198]. Such sediments are net sources of carbon to the atmosphere [199] that are prone to phosphorus internal loading [200] and the collection of microplastics [201].

**Figure 11:**
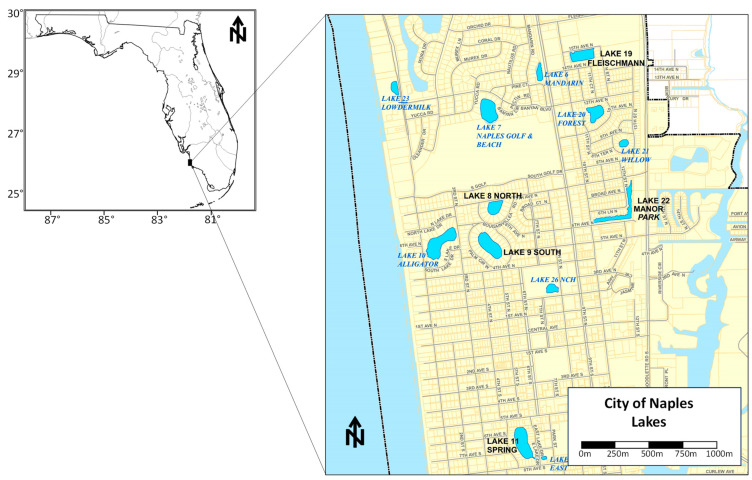
Location of the five urban wet stormwater ponds in the City of Naples, Florida, from which a total of 51 sediment cores were sampled and analyzed for Hg. Note: ponds are referred to as “lakes” and are numbered as well named.

**Figure 12:**
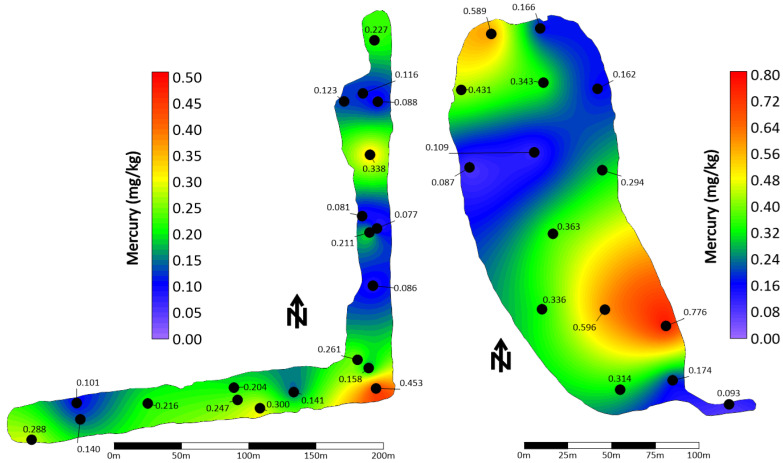
Mercury in sediment in Lakes 11 (**right**) and 22 (**left**). Sediment core locations (closed black circles) and Hg concentrations at those locations are represented on the maps as well as the mercury interpolated over space for each pond. Note the different mercury scales for the two ponds.

Besides sediment analyses mainly performed for dredging purposes [202,203,204,205,206], there is no published work of Hg accumulation in stormwater pond sediments. Hence, Hg analyses are presented from five detention ponds located in the City of Naples, Florida, from the aforementioned reports which include the concentration of Hg found in the organic portion of the sediment (i.e., not including the flocculate layer, which was discarded in the field) (Figure 11). Sediment was sampled with a handheld gravity coring device at various locations of the pond to uniformly cover the entire benthic area (Figure 12).

Once a sediment core was placed on the boat deck, it was extruded from its clear acrylic core barrel (inner diameter ranging from 5.08 cm to 8.89 cm) from the bottom up. The organic portion of the sediment was then visually separated from the rest of the sediment and placed in a Ziplock™ gallon bag, which was then stored in a cooler. In the laboratory, the sediment content was homogenized by hand in the bag and sent to a NELAC certified laboratory for metal analyses, including Hg, using the EPA 7471 Hg analysis method (Pace Analytical, Pompano Beach, FL, USA) and EPA 1631 (Florida International University, Bioinorganic and Environmental Analytical Facility, BEAF, Miami, FL, USA). The Hg concentrations varied spatially within each pond (Figure 12) and across them (Table 6). Hg concentrations ranged from 0.009 to 0.776 mg/kg, with an average of 0.18 7± 0.166 mg/kg.

**Figure 13:**
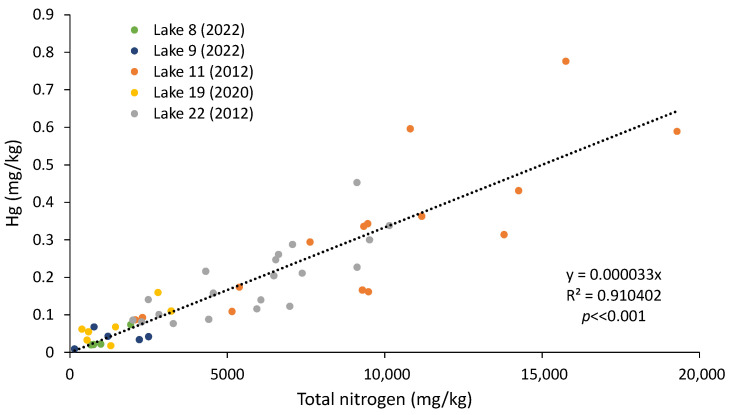
Relationship between Hg and total nitrogen in stormwater pond sediments.

**Figure 14:**
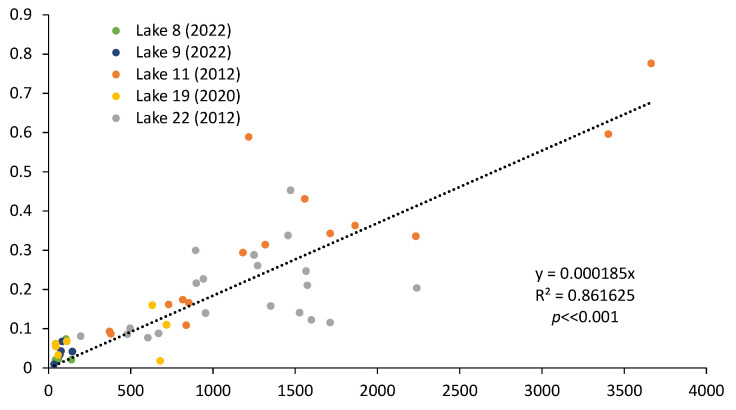
Relationship between Hg and total phosphorus in stormwater pond sediments.

There was a strong relationship between Hg and total nitrogen (TN, Figure 13) as well as total phosphorus (TP, Figure 14), suggesting onsite Hg methylation (MeHg) by the bacteria and algae living in the sediment [5,207,208,209,210,211]. TN and TP were also correlated with the organic content of the sediment for the samples for which this attribute was determined (R^2^ of 0.88 and 0.84, respectively, *p* << 0.001).

## 6. Hg Concentrations in Sediment, Surface Water, and Groundwater in Southern Florida

### 6.1. Atmospheric Hg Investigations in Southern Florida

In a study of the sources of atmospheric Hg sources in the atmosphere of Broward and Dade counties in south Florida, Graney et al. [212] found that of the average Hg concentration measured at 16.3 pg/m^3^, approximately 12 ± 2% originated in Saharan dust. This is 1.9 ± 0.4 pg/m^3^ based on conditions during the sampling periods.

Land use practices, and both natural and man-made fire, likely contribute to the movement of Hg in and out of the Everglades soils, hydraulically connected surface and groundwater, plants, and downwind regions in southern Florida.

**Figure 15:**
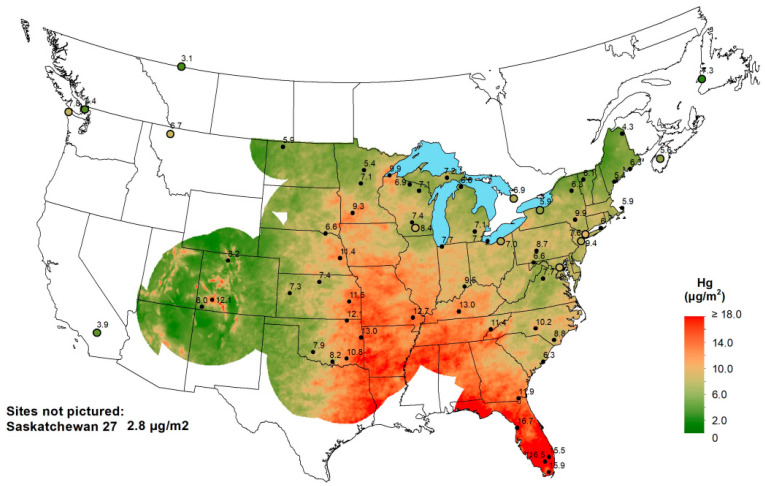
Measured annual wet deposition of mercury including three stations in southern Florida [213].

Wet deposition of Hg is monitored at three stations in southern Florida, with funding from the National Atmospheric Deposition Program ([213]; Figure 15). Note that the three values measured for the year 2021 averaged 16.0 μg/m^2^ and are among the highest values observed in the United States. Based on the combined land area of Monroe, Dade, Broward, Palm Beach, Hendry, Collier, and Lee counties, the wet deposition of Hg in southern Florida in 2021 was 411.4 kg. This value represents the combination of several sources contained within Table 7, which contains the influx factors. The overall distribution of Hg deposition as wet deposition does appear to follow the general pattern of Saharan dust distribution over the southeastern United States, particularly during the summer months [154].

**Figure 16:**
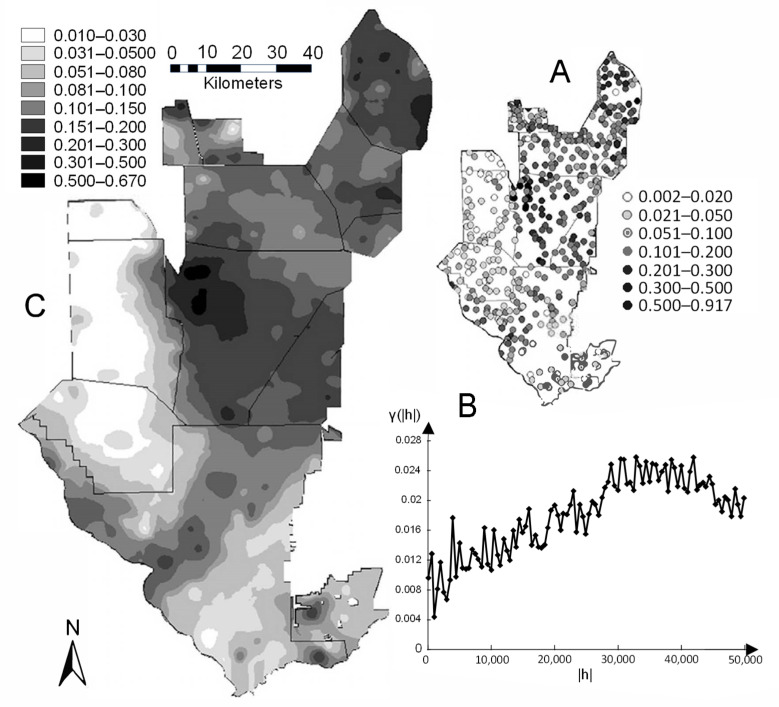
Total Hg per mass (THgM, mg/kg) showing (**A**) point observations, (**B**) modeled semivarigrams (500-m lags), and (**C**) an ordinary kriging prediction map (from Cohen et al. [137]).

The presence of Hg as wet deposition in Florida has been studied by Guentzel et al. [214] and Dvonch et al. [215] in the past. They have described some of the sources and effects on concentration. The high rate of Hg deposition in southern Florida was confirmed using measurements made on the soils of the Everglades by Cohen et al. [137] (Figure 16). The organic soils of the Everglades tend to sequester the deposited Hg and bind it into the soils. Some Hg leaves via evasion and into surface water and groundwater (Figure 7). However, wildfires (muck fires) tend to create major evasion events, thus returning the Hg to the atmosphere. While the emphasis of this discussion has been on wet deposition, dry deposition is also a factor within the context of Hg influx in southern Florida [69,132,216]. The dry deposition of Hg in southern Florida is also impacted by the Saharan dust influx. No reasonable estimate of dry deposition could be obtained within the context of total deposition.

### 6.2. Sources and Sinks of Hg in Southern Florida: A Proposed Mercury Budget

The largest reservoir of Hg in southern Florida is the organic soils of the Everglades peatlands (Figure 16). Organic sediments, particularly modern peats and ancient coal deposits are globally known as areas of heavy metals enrichment, especially Hg [72,95,217]. The primary source of Hg accumulation is atmospheric fallout caused in the past by volcanic inputs, with increased input during the last 700 years from anthropogenic sources, such as the burning of coal and peat. Saharan dust is also known to contain Hg and was and still is a major contributor to Hg accumulation in the southern Florida environment.

The enrichment of the Everglades peats is the likely result of between 3000 and 8000 years [140,141,142,143,144,145,146,147,148,149] of atmospheric deposition [91,92], with the concentration enrichment caused by fire-induced remobilization and redeposition [218,219,220], along with oxidation and compaction of the soil media [163].

Southern Florida has a very high atmospheric Hg accumulation rate of 25 μg/m^2^/y [207]. The authors have stated that local sources of Hg, including medical, municipal, and industrial incinerators, landfills, power plants, and urban activities, are responsible for this high rate. They also include global sources.

Since the Everglades peat deposit is the largest reservoir of legacy Hg in southern Florida, any issue that increases the outflux and mobilization of Hg away from the peaty soils is of major concern. Flower et al. [220] hypothesized that climate change-induced drought could increase the frequency of muck fires by 49%, which could mobilize more Hg in the atmosphere to be deposited in the downwind direction to the northwest and west, perhaps enriching soils and vegetation in areas distant from the Hg source (Everglades). The total original reservoir of mercury in the Everglades peatland is estimated to be approximately 42.3 metric tons (or higher) (Section 4.2). Aich et al. [163] reported that the total volume of peat in the Everglades was approximately 7 × 10^9^ m^3^ in approximately 1880 and reduced to approximately 3 × 10^9^ by 2013. If the original reservoir of mercury was 42.3 metric tons, it has been depleted by 65.7% in the last 133 years from 1880 to 2013, causing a discharge to the environment (principally the atmosphere) of 28.6 metric tons. If the loss of peat is averaged over those 133 years and is used as a proxy for Hg remobilization, then the annual rate of atmospheric discharge could be as high as 0.21 metric tons per year, or 215 kg/y.

Cohen et al. [137] suggest that anthropogenic Hg emissions in southern Florida have declined from 3000 kg/y in 1991 to 250 g/y in 2000. While Liu et al. [162] calculated that the annual deposition rate across the Everglades was <140 kg/y, Cohen at al. [137] concluded that there was no evidence that total Hg concentrations or soil mass are declining. A study suggested that source atmospheric Hg is likely based on both general atmospheric aerosol deposition from Saharan dust, with a possible enrichment by Everglades vegetation and/or muck fires [219]. Therefore, if muck fires continue to increase in frequency, the annual rate of Hg recycling from the Everglades may rise in time.

As part of the Hg budget in southern Florida, the losses of Hg from the ecosystem are mainly via wind transport that occurs during strong winter frontal systems that produce northeast winds across the entire peninsula. However, during the summer season, when Saharan dust movement is greatest, onshore winds during part of the day on both the east and west coasts of Florida tend to trap gaseous and particulate Hg on the land mass and in the atmosphere. Often, the Hg is incorporated into rainfall.

The principal sink of Hg is deposition on soils and plants. Organic soils tend to sequester the Hg, so wetland areas are permanently enhanced with Hg as well as other metals. The only discharge occurs during fires that invade wetlands (e.g., muck fires in the Everglades). Hg deposition on vegetation is a more complex matter. Greenplate et al. [187] recently found that 90% of the Hg found on the leaves of the wetland plant willow is surface deposited and washed off using deionized water. There was 8.8 times more Hg in the wood of the tree compared to that incorporated into the leaf matter. There has been no systematic investigation of Hg in plants in southern Florida. Based on the literature, Hg can remain within the plant wood or can be evaded from the leaves in certain cases (summarized by Mason [152]). In deciduous trees, Hg incorporated or surface-adsorbed on the leaves is recycled into the soils, where it may be permanently deposited or enters the groundwater or surface water in a dissolved form. A survey of metals concentrations in soils throughout Florida by Chen et al. [153] found that Hg varied from 0.00075 to 0.0396 mg kg^−1^, with the highest concentrations found in organic soil types. As observed in Figure 14, southern Florida has the highest concentration of Hg in rainfall in the United States on an annual basis.

Elevated concentrations of Hg have been found in estuarine sediments in southern Florida as well as mangrove peat deposits [221]. Kannan et al. [222] documented the total Hg and methylmercury in water, sediments, and fish in southern Florida estuaries.

## 7. Health Risk Aspects of Hg Exposure

### 7.1. Public Health Perspective of Mercury Exposure

Hg in the environment poses risks to the health of humans and wildlife, as described in the Global Mercury Assessment (GMA) [223] and accompanying documentation [223]. Hg toxicity has been a significant global health issue for over 2000 years [8,80]. The toxicity of Mg and its adverse impacts have been documented in ancient times, as recorded by Hippocrates (460–37 B.C.), Pliny the Elder (23–79 A.D.), Galen (131–200 A.D.), Avicenna (980–1037 A.D.), and Paracelsus (1493–1541 A.D.) [224,225]. The U.S. Public Health Service in 1941 documented the poisoning of workers exposed to Hg in the hat industry that resulted in the colloquial statement “mad as a hatter”. That hazard, as well as Hg exposure concerns in mining, thermometer/barometer manufacturing, and laboratory incidents, has been eliminated or largely reduced over the years [80].

In modern times, the most relevant exposure pathways for Hg to enter the human body in the general population are the breathing of contaminated air and the consumption of contaminated fish and shellfish, or other foods to a lesser extent [224,226]. The most toxic form of Hg in food is methylmercury. Hg can also enter the body via drinking water and can be absorbed into the body from oral or dermal contact with contaminated soils, which is a potential problem for children [6].

Depending on the exposure circumstances, Hg can exhibit a variety of human health impacts [227,228]. Sufficiently elevated blood concentrations can potentially result in myocardial infarction [229], reproductive and endocrine issues [230], increased colorectal cancer risk [231], and a possible association with increases in type 2 diabetes [232]. Multiple epidemiological studies in e.g., Minamata (Japan), the Seychelle Islands, and the Faroe Islands, have shown that the developing nervous system is an especially sensitive target of methylmercury intake during pregnancy [233,234]. The significant differences in outcomes of these studies may be explained by the varied attention to potential confounders such as nutritional benefits of fish-based omega-3 fatty acids, concurrent exposure to other contaminants in fish tissue (e.g., selenium, PCBs), and social aspects of child development. As noted in a 2019 study specific to the consumption of fish by pregnant women in south Florida, Schaefer et al. [235] emphasized that “Educational efforts must provide a balanced approach to include the benefits of fish consumption while minimizing risk by avoiding locally caught seafood or fish species known to contain high levels of Hg”.

### 7.2. Exposure to Hg in Soils, Surface Water, and Drinking Water in Southern Florida: Health and Regulatory Perspectives

Although soil-based Hg may be a significant source of indirect human exposure (e.g., inhalation of off-gassed Hg, and runoff to waterways with subsequent consumption of contaminated fish and shellfish), direct human exposure to Hg in soils via ingestion, inhalation, and dermal contact generally is not a common route of significant exposure. However, in southern Florida, with the noted legacy reservoir of Hg in Everglades mucky soils, farming, recreational, and other activities may contribute in a notable way to the direct and indirect intake of Hg from soil. The Florida Department of Environmental Protection [236] has established health protective levels for mercury in soil, which are shown in Table 8. 

In cooperation with the USEPA, the FDEP has established a statewide Hg Total Daily Maximum Load (TMDL) for fresh and marine surface waters. The TMDL aims to assist in the identification of impaired waterbodies and to facilitate reductions in pollutant loads so that a return to previous designated uses is possible [237]. The Hg TMDL is unique in that it is not based on actual surface water Hg concentrations. In fact, although water quality standards are in place for Florida (e.g., Class III standards for fish consumption and recreation for fresh (≤0.012 μg/L) and marine (≤0.025 μg/L) waters), there are no waterbodies in Florida that have been identified as impaired based solely on Hg concentrations in the water. Rather, the TMDL is based on exceedances of Florida Department of Health (FDOH) guidelines regarding the consumption of fish and shellfish [237]. As detailed in the Mercury TMDL Final Report [237], waterbodies are generally listed as impaired only if FDOH fish consumption advisories have been established due to elevated levels of Hg in edible tissues of species in that waterbody.

A search of data for Hg in drinking water in southern Florida shows very low concentrations in public drinking water. In virtually all cases, it occurs below not only the Federal and Florida drinking water standard of 2 μg/L (2 ppb), but below the detection limits of various analytical methods (e.g., less than 0.1 μg/L). Some private wells producing water from shallow, unconfined aquifers can become contaminated with dissolved Hg, especially where located near domestic landfills containing old solid waste deposited before modern Hg control laws were passed.

Thus, although no reports of significant adverse human health effects associated with direct exposure to soil, surface water, or drinking water were found, regulations are in place for each of those media to ensure that the potential health hazards are minimized.

### 7.3. Exposure to Hg in Ambient Air: Health and Regulatory Aspects

Although occupational standards exist for worker exposure to Hg in air (e.g., OSHA 8 h TWA of 0.1 mg/m^3^), protective levels for exposure to Hg in ambient outdoor air are not available. Potentially applicable health-protective guidelines for Hg in the air include the USEPA inhalation reference concentration (RfC) for elemental Hg at a concentration of 3.0 × 10^−4^ g/m^3^, as well as the residential and commercial Regional Screening Levels (RSLs) that are based on the RfC. The residential indoor air RSL is 0.31 μg/m^3^, and the commercial RSL is 1.3 μg/m^3^ [10]. All of the southern Florida atmospheric Hg concentrations discussed previously in Section 6.1 and elsewhere are considerably less than the health-protective guidelines for inhalation exposure.

### 7.4. Hg Bioaccumulation in Fish and Animals

**Figure 17:**
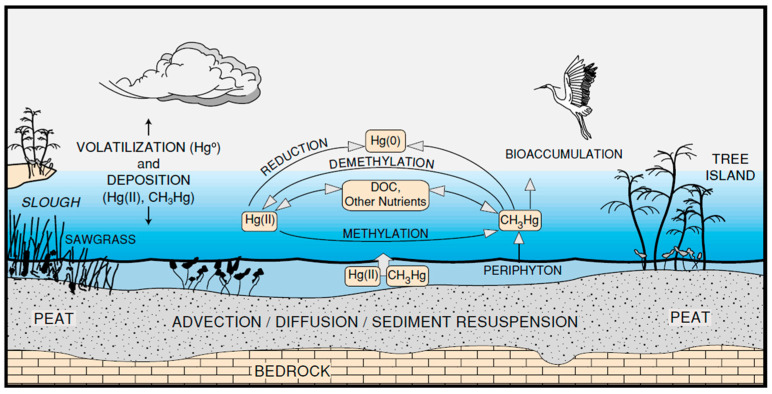
Food web bioaccumulation of methylmercury in fish, birds, and other organisms. The methylmercury origin is located at sediment–periphyton interface (from Krabbenhoft et al. [5]).

Hg accumulation in plants and animals tends to biomagnify up the food chain in both marine and freshwater environments [101,102,111,222,238,239,240]. Commonly, elemental and particulate Hg are initially deposited in plants and soils and undergo the process of methylation, which allows the easy uptake of organic Hg into the food chain [4,41,42,241,242]. Studies conducted in the Everglades documented the processes by which Hg is methylated and demethylated, as well as how it enters the food chain [5,88,208,209,210,211]; Figure 17).

**Figure 18:**
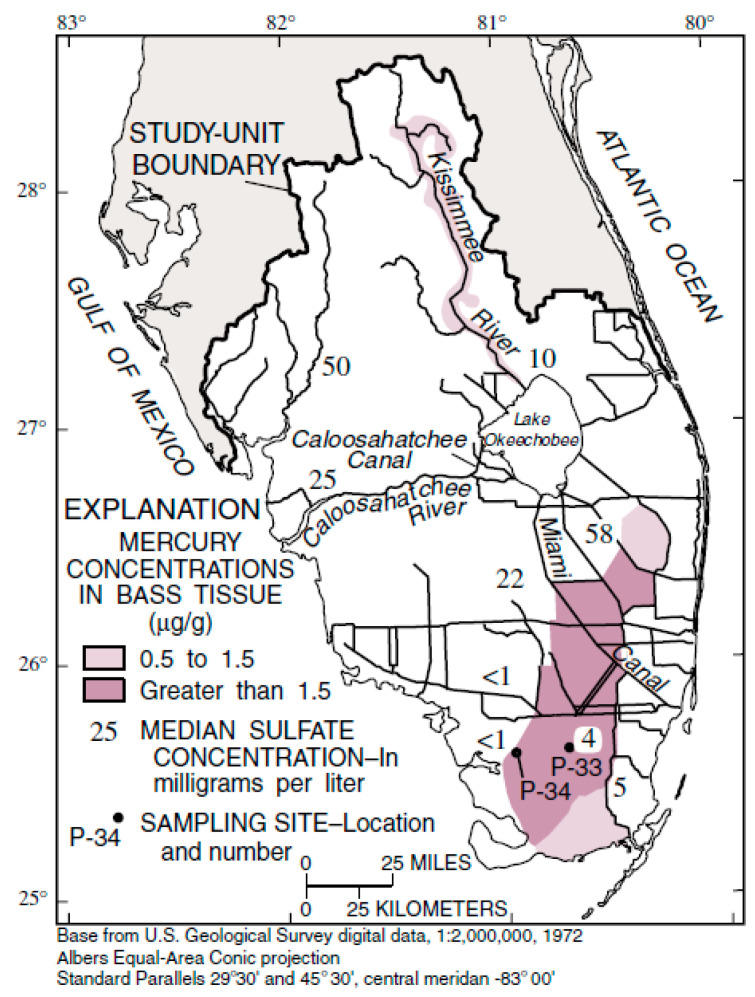
Hg concentrations in bass liver tissue in southern Florida [247].

The bioaccumulation of methylmercury in the Everglades biota shows the accumulation beginning with small fish, such as mosquitofish (e.g., Gambusia holbrooki; [209,211]), and then larger fish, such as largemouth bass [209,211,243], to wading birds, such as egrets [244,245], and ultimately to an apex predator such as the Florida panther [246]. In one study, it was reported that the Hg in game fish in the Everglades exceeded the recommended human consumption guideline of 1.5 μg/g (mg/kg), with a maximum reported detection of 4.4 μg/g in edible portions of largemouth bass (Figure 18; [247,248]). As illustrated in Figure 17, elevated concentrations of mercury in bass livers covered a wide portion of the Everglades and were associated with the sulfate concentration, which is involved in the methylation process.

Since largemouth bass commonly are eaten by fisherman and their families, the State of Florida has issued health warnings to try to eliminate the severe impacts of consumption of fish tainted with Hg (Florida Department of Health [249]. The Hg in bass issue is not limited to the Everglades, but also impacts Lake Okeechobee and rivers and streams in southern Florida.

## 8. Conclusions

Southern Florida has the highest Hg concentration in the atmosphere within the continental United States, with an average annual wet deposition rate (2021) of 16.0 μg/m^2^. The total deposition of all forms of new mercury is estimated to be 411.2 kg/y for the area including Monroe, Dade, Broward, Palm Beach, Hendry, Collier, and Lee counties, which is an area of 25,720 km^2^. The highest influx of naturally occurring Hg is from Saharan dust, marine evasion, and global aerosols, which in combination contribute between 257.1 and 514.2 kg/y. Another major contributor of Hg to the atmosphere of southern Florida is the remobilization of legacy Hg from the Everglades. Prior to the impacts of man on the Everglades, it contained a reservoir of Hg of an estimated 42.3 metric tons. This mercury was bound within the organic soils (peat) which have had a reduction in volume of approximately 65.7%, resulting in a discharge to the environment (principally the atmosphere) of 28.6 metric tons of recycled legacy Hg. This remobilization has occurred over a period of 133 years, which would make its annual contribution to the Hg budget of up to 215 kg/y. Anthropogenic sources of Hg within southern Florida include incinerators (waste to energy), other incinerators (medical waste, crematories), cement plants, and motor vehicle fuel emissions. These sources of Hg contribute 365.2 kg/y. Surprisingly, emissions of Hg from the five large natural gas-fired power plants in southern Florida are trivial compared to other Hg sources (<2 kg/yr). The range in total additional or recycling of mercury to the southern Florida environment ranges from 995.9 to 1253 kg/y.

A key unresolved question is where Hg which enters the southern Florida environment is being deposited, or what the primary sinks are. Some of the atmospheric Hg does leave during frontal systems during the winter months when strong northeast winds move it into the Gulf of Mexico or towards Mexico. However, Hg entering the system from Saharan dust and marine evasion occurs primarily during the summer months and is deposited mostly via wet deposition throughout the region. During a large part of the summer, winds blow onshore on both the east and west coasts, thereby trapping Hg in the atmosphere for atmospheric removal by rainfall and/or particulate deposition. A recent study of willow trees in southwest Florida found that 90% of Hg found on leaves was surface deposited and not incorporated into the leaf tissue, which supports a significant rate of atmospheric deposition. The sink for Hg deposition in southern Florida requires a major research initiative.

The high rates of Hg deposition, and the legacy content in southern Florida environmental media, combined with potential health effects via multiple routes of exposure, suggest that health impacts may be occurring without adequate research to define and quantify a link to this potentially significant health hazard.

## Figures and Tables

**Table 1:** Annual deposition rates for Hg in the Pinet and Estibere peat bogs (modified from Enrico et al. [72]). All values in μg m^−2^ y^−1^. CE means Common Era, similar to AC or BC.

**Peat Bog**	**8008–1000 CE**	**500–1500 CE**	**1760–1880 CE**	**1971–2001 CE**	**2001–2011 CE**
Pinet					
HgAR_total_	1.5 ± 1.0	1.7 ± 0.1	6.3 ± 2.4	40 ± 6	29 ± 3
HgAR_dry_	1.1 ±0.4	1.22 ± 0.07	4.7 ± 1.8	36 ± 7	24 ± 2
HgAR_wet_	0.4 ± 0.2	0.46 ± 0.02	1.8 ± 0.7	5.1 ± 2.9	4.5 ± 0.9
		**800–1500 CE**	**1760–1880 CE**	**1946–1967 CE**	**1990–2011 CE**
Estibere					
HgAR_total_	NA	2.4 ± 0.5	6.0 ± 1.3	24 ± 2	9.2 ± 3.5
HgAR_dry_	NA	1.3 ± 0.3	3.4 ± 0.8	14 ± 1	5.2 ± 2.0
HgAR_wet_	NA	1.1 ± 0.3	2.6 ± 0.6	11 ± 1	4.0 ± 1.5

**Table 2:** Chemical Properties of Hg.

Property	Number or Description
Atomic number	80
Atomic weight (std)	200.592 (3)
Oxidation states	−2, +1, +2
Atomic radius	1.51 angstrom
Covalent radius	1.31 ± 0.05 angstrom
Van der Waals radius	1.55 angstrom
Electronegativity	Pauling: 2.00
Melting point	234.3210 K
Vapor pressure (std)	0.00243 atm
Heat of fusion	2.29 kJ/mol
Heat of vaporization	59.11 kJ/mol
Molar heat capacity	27.893 J(mol*K)

**Table 3:** Hg concentration in various carbonate rocks (in μg/kg). Note that measurement error in these concentrations is commonly not reported and is generally greater in the older literature based on modern improvements in detection.

Location	No. Samples	Min.	Max.	Avg.	Reference
Unknown location	1	-	-	33	Stock and Cucuel [120]
Germany	14	28	220	66	Heide et el. [121]
Russian Platform (argillaceous marls)	19	10	90	31	Ozerova and Aidin’yan [122]
	1	10	8000	-	Abuev et al. [123]
Crimean highlands (limestones)	8	100	6400	2300	Bulkin [124]
Crimean highlands (marls)	5	500	5000	1000	Bulkin [124]
Donets Basin	314	<100	10,000	900	Karasik and Goncharov [125]
Southern Ferghana (limestones and dolomites)	22	20	150	75	Nikiforov et al. [126]
Northeast Yakutia	26	<2	70	18	Nekrasov and Timofeeva [127]
Vietnam (marble)	1	-	-	500	Aidin’yan et al. [128]
India (beachrock)	25	0.06	0.31	0.17	Sahayam et al. [129]

**Table 4:** Hg concentrations measured in carbonate rocks of Early Eocene to Miocene in age from southern Florida and adjacent counties by the Florida Geological Survey. The detection of mercury in rock samples can be problematic based on the methods used and these reported values appear to be low compared to more recent investigations (bls is defined as below land surface and BDL stands for Below Detection Limits).

Well No.	Depth Minimum (m bls)	Depth Minimum (m bls)	Formation	County	Hg (μg/kg) Hg-FIMS
W-17001	154.9	155.2	Hawthorn	Highlands	5
W-17001	168.4	168.6	Hawthorn	Highlands	8
W-17001	187.8	188.1	Hawthorn	Highlands	12
W-17001	201.7	202.1	Suwannee	Highlands	BDL
W-17001	208.2	211.3	Suwannee	Highlands	BDL
W-17001	210.7	211.3	Suwannee	Highlands	BDL
W-17001	214.6	215.2	Ocala	Highlands	BDL
W-17001	218.3	218.9	Ocala	Highlands	5
W-17001	225.3	226.8	Ocala	Highlands	5
W-17001	236.6	237.0	Ocala	Highlands	BDL
W-17001	247.0	247.4	Ocala	Highlands	BDL
W-17001	261.3	281.9	Ocala	Highlands	BDL
W-17986	309.5	309.8	Hawthorn	Palm Beach	13
W-17986	310.7	311	Hawthorn	Palm Beach	8
W-17986	311.6	311.9	Hawthorn	Palm Beach	11
W-17986	318	318.3	Hawthorn	Palm Beach	145
W-17986	318.9	319.5	Hawthorn	Palm Beach	7
W-17986	319.6	320.1	Hawthorn	Palm Beach	9
W-17986	344.5	344.8	Avon Park	Palm Beach	9
W-17986	345.4	345.7	Avon Park	Palm Beach	6
W-17986	352.1	352.4	Avon Park	Palm Beach	10
W-17986	356.7	357.0	Avon Park	Palm Beach	76
W-18253	266.5	266.8	Suwannee	Glades	BDL
W-18253	267.4	267.7	Suwannee	Glades	104
W-18253	268.6	268.9	Suwannee	Glades	BDL
W-18253	303.0	303.4	Ocala	Glades	BDL
W-18253	304.3	304.6	Ocala	Glades	BDL
W-18253	306.4	306.7	Ocala	Glades	51
W-18253	397.6	397.9	Avon Park	Glades	33
W-18253	399.1	399.4	Avon Park	Glades	43
W-18253	400.6	400.9	Avon Park	Glades	15
W-18255	176.8	178.4	Ocala	Okeechobee	BDL
W-18255	209.1	209.5	Ocala	Okeechobee	BDL
W-18255	210.4	210.7	Ocala	Okeechobee	BDL
W-18255	225.6	234.8	Ocala	Okeechobee	BDL
W-18255	243.9	253	Ocala	Okeechobee	BDL
W-18255	260.7	269.8	Avon Park	Okeechobee	19
W-18256	194.5	184.7	Hawthorn	Martin	23
W-18256	196	196.2	Hawthorn	Martin	19
W-18256	243.3	243.6	Ocala	Martin	20
W-18256	244.5	244.8	Ocala	Martin	24
W-18256	245.4	245.7	Ocala	Martin	20
W-18256	278.7	279	Ocala	Martin	BDL
W-18256	279.9	280.2	Ocala	Martin	6
W-18256	284.2	284.5	Avon Park	Martin	BDL
W-18256	286.0	286.3	Avon Park	Martin	BDL
Average					15.3
Maximum					145
Minimum					BDL

**Table 5:** Domestic and industrial uses of Hg compounds as preservatives, fungicides, mildew control, and bacterial control (from USEPA [80], not complete list). The question mark means that there data are not available.

Compound	Use	Application Concentration (μg/g)
Phenylmercuric acetate	Preservation	45–250
Phenylmercuric acetate	Mildew control	3500–15,000
Phenylmercuric oleate	Fungicide on furniture	500–5000
Phenylmercuric hydroxide	Bacterial preservative (paint)	5000 (est.)
Phenylmercuric acetate	Bacterial preservative (cement)	1600 (est.)
Phenylmercuric acetate	Fungicide (cement and plaster preservation)	45–200
Phenylmercuric acetate	Fungicide (cement and plaster after application)	3500–15,000
Mercuric oxide	Marine antifouling coating	?
Phenylmercuric oleate	Bacterial preservative (pint)	1500 (est.)
Chloromethoy-acetoxymercuipropane	Mildew control (paints, stains, varnishes)	?
Di (phenylmercury) dodcylsuccinate	Mildew control (paints, stains, varnishes)	3750
Phenylmercuric acetate	Mildew control (paints, stains, varnishes)	1500 (est.)
Phenylmercuric borate	Mildew control (paints, stains, varnishes)	1500 (est.)
Phenylmercuric hydroxide	Mildew control (paints, stains, varnishes)	4500–9000
Phenylmercuric oleate	Mildew control (paints, stains, varnishes)	?
Phenylmercuric oleate	Bacterial preservative (marine paint)	1500 (est.)
Chloromethoy-acetoxymercuipropane	Mildew control (paints, stains, varnishes)	3570
Di (phenylmercury) dodcylsuccinate	Mildew control (paints, stains, varnishes)	3570
Phenylmercuric acetate	Mildew control (paints, stains, varnishes)	1500 (est.)
Phenylmercuric borate	Mildew control (paints, stains, varnishes)	1500 (est.)
Bacteriostatic (paint)	Mildew control (paints, stains, varnishes)	1500 (est.)
Phenylmercuric acetate	Bacteriostatic (paint)	3000 (est.)
Phenylmercuric propionate	Bacteriostatic (latex paint on asbestos)	5000 (est.)
Chloromethoxypropylmercuric	Bacteriostatic (latex paint on asbestos)	5000 (est.)
Phenylmercuric acetate	Bacteriostatic (latex paint)	5000 (est.)
Di (phenylmercury) dodcylsuccinate	Wallpaper coating	?
Phenylmercuric acetate	Fungicide (fabrics and textiles)	25–225
Phenylmercuric borate and chloride	Fungicide (industrial fabrics and textiles)	26.5
Phenylmercuric oleate	Fungicide (awnings, sail covers, boat covers)	2000–2800
Phenylmercuric acetate	Mold resistant paper	150–225
Phenylmercuric acetate	Fungicide (plastics surface)	150–225
Phenylmercuric borate	Fungicide (plastics surface)	50
Phenylmercuric propionate	Fungicide (plastics surface)	?
Phenylmercuric borate	Fungicide (plastics-polystyrene)	50
Phenylmercuric propionate	Bacteriostatic surface coating (plastics-PVC)	?
Phenylmercuric hydroxide	Bacteriostatic (vinyl)	?
Phenylmercuric acetate	Fungicide (rubber)	125–225
Phenylmercuric borate	Fungicide (rubber)	50
Phenylmercuric acetate	Bacteriostatic (floor wax)	High (undiluted)
Phenylmercuric acetate	Bacteriostatic and fungicide (tanneries)	305
Ethylmercury phosphate	Mold control (wood)	150–300
Phenylmercuric acetate	Mold control (wood)	1600
Phenylmercuric hydroxide	Mold control (wood)	High
Phenylmercuric lactate	Mold control (wood)	High
Mercuric chloride	Rot control (fence posts)	High (soak)
Phenylmercuric oleate	Rot control (fence posts)	High (soak)
Phenylmercuric borate	Dental tool disinfection	High
3-(hydroxymercuri)-4-nitro-o-cresol	Dental tool disinfection	High

**Table 6:** Average Hg concentrations and variations across stormwater ponds. * Note that all values recorded were above the MDL of 0.016 mg/kg besides Lake 9, with one value being 0.0092 mg/kg. For this sample, the value of MDL/2 (0.008 mg/kg) was used for the statistical computations.

		Hg (mg/kg)			
Lakes	Mean ± S.D. (n)	Min	Max	Range	EPA Method
8	0.038 ± 0.031 (3)	0.020	0.074	0.054	7471
9	0.039 ± 0.022 (6)	0.009 *	0.068	0.059	7471
11	0.322 ± 0.206 (15)	0.087	0.776	0.689	1631
19	0.072 ± 0.048 (7)	0.018	0.160	0.142	7471
22	0.193 ± 0.101 (20)	0.077	0.453	0.376	1631
Overall	0.187 ± 0.166 (51)	0.009	0.776	0.767	

**Table 7:** Estimated atmosphere mercury influx for southern Florida. Where a question mark occurs, we could not find data to populate the table.

Sources	Approximate Annual Inflows/kg yr^−1^	Notes	References
Sea spray/ocean flux/evasion excluding the Everglades/Saharan dust and aerosols	257.1–514.2	It is not possible to clearly quantify each of these sources	National Atmospheric Deposition Program, 2021; Section 4.3
Soil/dust/local sources	?	Insufficient data	
Cement plant stack discharge	150.6		FDEP, 2023
Electrical power generation plants (natural gas fired)	0 ^a^	Most measurement below the threshold for reporting	FDEP, 2023
Incinerators/waste to energy	204.1		FDEP, 2023
Incinerators/medical/crematories	159.7+		FDEP, 2023
Fuel emissions from vehicles	1.4	Calculated	Section 5.7
Plant transpiration	?	Contradictory data	Mason, 2009
Municipal landfalls/gas discharge	4		Section 5.6
Minor: asphalt plants, soil amendments evasion	<1	Not fully characterized	FDEP, 2023
Remobilization via wildfire and peat loss in Everglades peatland	215	Based on loss of peat over 133 years	Section 6.2
Remobilization in sugarland controlled fires	?	Insufficient data (could be grouped into peat loss)	None available
Approximate Totals	991.9–1249		

^a^ data below the threshold for reporting.

**Table 8:** Health protection maximum concentrations of Hg for residential and commercial land used established by the Florida Department of Environmental Protection.

	Residential Land Use mg/kg	Commercial/Industrial Land Use mg/kg
Mercury (Hg)	3	17
Methylmercury	1.1	6.1

## Data Availability

All data used or developed is contained within the paper.
